# Genetic compensation for cilia defects in *cep290* mutants by upregulation of cilia-associated small GTPases

**DOI:** 10.1242/jcs.258568

**Published:** 2021-07-22

**Authors:** Magdalena Cardenas-Rodriguez, Christina Austin-Tse, Judith G. M. Bergboer, Elisa Molinari, Yuya Sugano, Ruxandra Bachmann-Gagescu, John A. Sayer, Iain A. Drummond

**Affiliations:** 1 Department of Medicine, Nephrology Division, Massachusetts General Hospital, 149 13th Street, Charlestown, MA 02129, USA; 2 Human Molecular Genetics Laboratory, Institut Pasteur de Montevideo, Mataojo 2020, 11400 Montevideo, Uruguay; 3 Department of Pathology, Massachusetts General Hospital, 185 Cambridge St, Boston, MA 02114, USA; 4 Cergentis BV, Yalelaan 62, 3584 CM Utrecht, The Netherlands; 5 Translational and Clinical Research Institute, Faculty of Medical Sciences, Newcastle University, Newcastle NE1 3BZ, UK; 6 Institute of Anatomy, University of Zurich, Winterthurerstrasse 190, 8057 Zurich, Switzerland; 7 Institute of Medical Genetics, University of Zurich Wagistrasse 12, CH-8952 Schlieren, Switzerland; 8 Renal Services, The Newcastle upon Tyne Hospitals NHS Foundation Trust, Freeman Road, Newcastle NE7 7DN, UK; 9 Davis Center for Regenerative Biology and Aging, Mount Desert Island Biological Laboratory, Salisbury Cove, Bar Harbor, ME 04609, USA

**Keywords:** Cep290, NPHP6, Ciliopathy, Genetic compensation, Zebrafish, Kidney epithelial cell

## Abstract

Mutations in CEP290 (also known as NPHP6), a large multidomain coiled coil protein, are associated with multiple cilia-associated syndromes. Over 130 *CEP290* mutations have been linked to a wide spectrum of human ciliopathies, raising the question of how mutations in a single gene cause different disease syndromes. In zebrafish, the expressivity of *cep290* deficiencies were linked to the type of genetic ablation: acute *cep290* morpholino knockdown caused severe cilia-related phenotypes, whereas deficiencies in a CRISPR/Cas9 genetic mutant were restricted to photoreceptor defects. Here, we show that milder phenotypes in genetic mutants were associated with the upregulation of genes encoding the cilia-associated small GTPases *arl3*, *arl13b* and *unc119b*. Upregulation of UNC119b was also observed in urine-derived renal epithelial cells from human Joubert syndrome *CEP290* patients. Ectopic expression of *arl3*, *arl13b* and *unc119b* in *cep290* morphant zebrafish embryos rescued Kupffer's vesicle cilia and partially rescued photoreceptor outer segment defects. The results suggest that genetic compensation by upregulation of genes involved in a common subcellular process, lipidated protein trafficking to cilia, may be a conserved mechanism contributing to genotype-phenotype variations observed in *CEP290* deficiencies.

This article has an associated First Person interview with the first author of the paper.

## Abstract (precis)

**Summary:** The impact of a ciliopathy gene mutation depends on cell type, and can be compensated for by expression of other genes that function in the same cilia membrane protein delivery pathway.

## INTRODUCTION

Ciliopathies are a group of human genetic disorders caused by mutations in genes related to cilia function. Motile cilia are responsible for fluid propulsion and clearance in the lung, for instance (Lee and Ostrowski, 2021), whereas primary or non-motile cilia serve a sensory or signaling function (Reiter and Leroux, 2017). Motile ciliary defects cause primary ciliary dyskinesia (Lee and Ostrowski, 2021), whereas primary ciliopathies comprise a phenotypic and genetically diverse group of syndromes, ranging from isolated retinal dystrophy, as in Leber's congenital amaurosis, to syndromes in which multiple organs are affected, such as Joubert Syndrome (JBTS), Senior–Løken syndrome, Meckel syndrome (MKS), Bardet–Biedl syndrome (BBS) and nmephronophthisis (NPHP) (Reiter and Leroux, 2017). Nearly 200 genes have been associated with ciliopathies, and despite the heterogeneity in their phenotypes and etiology, several share common causal genes (Badano et al., 2006; Reiter and Leroux, 2017). Mutations in CEP290 (also known as NPHP6), a large multidomain coiled coil protein, have been associated with at least five different cilia-associated syndromes (Reiter and Leroux, 2017). Mutations in human CEP290 are commonly associated with Leber's congenital amaurosis, an isolated degenerative blindness (den Hollander et al., 2006), but can also lead to outcomes as severe as Meckel–Grüber syndrome (failed neural tube closure, encephalocele and perinatal lethality) (Baala et al., 2007; Frank et al., 2008). Intermediate phenotypes result in kidney cysts (juvenile nephronophthisis), as well as brain development and fiber tract abnormalities (JBTS) (Reiter and Leroux, 2017). CEP290 localizes to the centrosome/basal body and to the cilia transition zone in both motile and non-motile cilia where it regulates the selective trafficking of proteins into the cilia domain (Chang et al., 2006; Craige et al., 2010). *Drosophila* CEP290 has recently been shown to play an essential role in the initiation of cilia transition zone assembly (Wu et al., 2020). CEP290 also has an essential role in centriolar satellite function in which it regulates the assembly and disassembly of protein complexes that are transported toward the base of the cilia (Kim et al., 2008). To date, more than 130 mutations in *CEP290* have been linked to a spectrum of human ciliopathies, raising the question of how mutations in a single gene can cause so many different disease syndromes (Coppieters et al., 2010). Establishing a clear genotype-phenotype correlation in animal models has also been challenging, as allelic variation, as well as the manner in which *CEP290* expression is inhibited, influences resulting phenotypes. In *Cep290* mouse mutants, the *rd16* line lacking exons 35 to 39 (in-frame deletion of 897 bp) shows only retinal degeneration, whereas the *Cep290* knockout mouse showed retinal degeneration and also hydrocephalus, kidney cysts and cilia axoneme defects (Chang et al., 2006; Hynes et al., 2014; Rachel et al., 2015). These discrepancies are also observed in zebrafish models, in which acute *cep290* morpholino knockdown showed retinal defects and a range of cilia-related phenotypes, whereas genetic mutants induced by ethylnitrosourea or TALEN genome modification showed milder phenotypes restricted to photoreceptor defects (Baye et al., 2011; Lessieur et al., 2019; Sayer et al., 2006; Stawicki et al., 2016).

Several explanations have been proposed to explain the lack of consistency between genotype and phenotype in *CEP290* patients and animal models. One possibility is that different genetic backgrounds may contribute modifier genes that compensate or alter *CEP290* deficiency (Coppieters et al., 2010; Gorden et al., 2008; Lessieur et al., 2019; Ramsbottom et al., 2020; Rao et al., 2016). Another possibility is that basal exon skipping or nonsense-associated alternative splicing may promote bypassing *CEP290* mutations and the expression of functional CEP290 protein (Barny et al., 2019; Littink et al., 2010). CEP290 protein dosage; i.e. the amount of CEP290 protein retaining all or some of the full-length functionality, has also been proposed to determine the range and severity of *CEP290* deficiency phenotypes (Drivas et al., 2015). Despite these efforts to understand phenotypic variation associated with *CEP290* mutations, questions remain about mechanisms of *CEP290* phenotype expression (Barny et al., 2019; Littink et al., 2010). Several recent reports indicate that gene inactivation can activate genetic compensation mechanisms that contribute to phenotypic variability or the absence of phenotypes in diverse animal models (Buglo et al., 2020; Ma et al., 2019; Rossi et al., 2015; Serobyan et al., 2020). This genetic compensation response seems to rely on the presence of a premature termination codon in the mutated mRNA and sequence similarity between the mutated and the upregulated genes (El-Brolosy et al., 2019).

To determine whether genetic compensation may be implicated in *cep290* genotype-phenotype variation, we compared cilia-associated phenotypes in acute zebrafish morpholino knockdown and stable CRISPR/Cas9 *cep290* mutant zebrafish. We find that milder tissue-restricted phenotypes in *MZcep290* CRISPR/Cas9 zebrafish mutants compared to morphants is linked to an upregulation of genes encoding cilia proteins that mediate cilia lipidated protein transport, including *arl3*, *arl13b* and *unc119b*. Ectopic expression of *arl3*, *arl13b* and *unc119b* rescued Kupffer's vesicle cilia defects and partially rescued photoreceptor outer segment defects in zebrafish *cep290* morphant embryos. Upregulation of UNC119b was also observed in urine-derived renal epithelial cells from JBTS patients with *CEP290* variants, suggesting that genetic compensation by genes and proteins involved in a common subcellular process may be a conserved mechanism contributing to genotype-phenotype variations observed in patients with *CEP290* mutations.

## RESULTS

### Acute versus long-term loss of cep290 differentially affects cilia structure

**Fig. 1.**
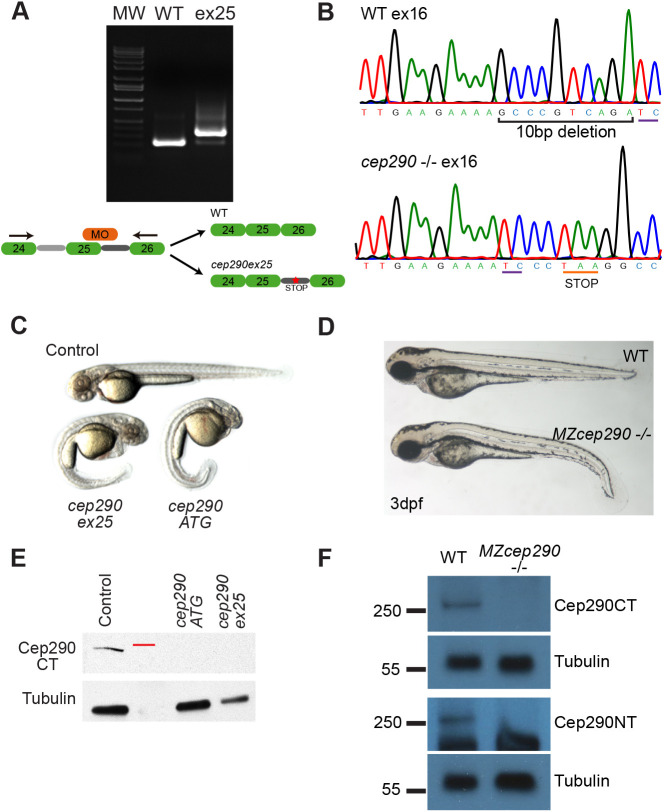
**Cep290 disruption causes ciliopathy in zebrafish.** (A) RT-PCR from representative single wild-type (WT) and *cep290ex25* morphant embryos. Cep290 morpholino oligonucleotide targeted to the exon 25 splice donor caused mispliced RNAs and intron retention. (B) Chromatogram of a CRISPR/Cas9-generated single representative genetic *cep290* mutant showing the 10-bp deletion in exon 16 that results in a premature stop codon. (C) Morphology of an uninjected embryo compared to *cep290ex25* and *cep290ATG* morphants at 2 dpf. (D) Morphology of wild-type and *MZcep290^fb208/fb208^* mutants (hereafter called *MZcep290*^−/−^ mutants) at 3 dpf. (E) Western blot showing loss of Cep290CT immunoreactivity in *cep290ATG* and *cep290ex25* morphants (pool of 40 48-hpf embryos). The position of the 250-kDa molecular weight marker is indicated in red. A tubulin antibody was used as a loading control (bottom). (F) Western blot showing loss of Cep290CT and Cep290NT immunoreactivity in *MZcep290*^−/−^ mutants. A tubulin antibody was used as a loading control. Experiments were replicated a minimum of three times.

To better understand how mutations impact CEP290 function, we reassessed the consequence of different *cep290* deficiencies in zebrafish. We used three different antisense morpholinos targeting the ATG translation initiation codon, previously published morpholinos targeting the exon 25 splice donor (Baye et al., 2011; Fig. 1) and an exon 42 splice donor (Sayer et al., 2006; Fig. S1) to acutely disrupt *cep290* expression. These exons were targeted, as misplicing generated out of frame exon skipping, predicted to introduce stop codons and premature translation termination. Injection of the *cep290* exon 25 splice donor morpholino (Baye et al., 2011) abrogated expression of wild-type *cep290* mRNA and induced expression of mispliced *cep290* RNAs, which were predicted to cause premature protein truncation (Fig. 1A). Additionally, we generated a stable *cep290* mutant using CRISPR/Cas9 in which a 10 bp deletion in exon 16 causes a frameshift and premature termination codon (allele designation *fb208*; Fig. 1B). Exon 16 was targeted based on high efficiency Cas9 cutting and introduction of N-terminal out-of-frame stop codons. Acute deficiency in *cep290* with morpholino injection produced canonical zebrafish ciliopathy phenotypes (Fig. 1C), such as body curvature, kidney cysts, otolith defects and left-right asymmetry, consistent with previous reports (Fig. S1A-D) (Baye et al., 2011; Sayer et al., 2006; Schäfer et al., 2008). On the other hand, *cep290* CRISPR/Cas9 mutant embryos at 3 days post-fertilization (dpf) showed a much weaker phenotype of mild ventral axis curvature with variable penetrance (20-50%) (Fig. 1D). Maternal zygotic (MZ) *cep290^fb208/fb208^* mutants (hereafter referred to as *MZcep290*^−/−^) did not exhibit kidney cysts, otolith or left-right asymmetry defects (Fig. S1D), in accordance with previous reports from the zebrafish *cep290 ^fh297^* line (Lessieur et al., 2019). Also, the majority of *MZcep290* mutant embryos (75%) that initially showed a mild axis curvature at 2-3 dpf completely recovered a straight body axis by 7-8 dpf (see below and Fig. 4A,B). For the rest of our studies, we focused on *MZcep290*^−/−^ embryos, which did not show strong evidence of ciliopathy. Using two antibodies specific to the C and N terminus of the zebrafish Cep290 protein (Cep290CT and Cep290NT, respectively), western blotting confirmed that the full-length Cep290 protein is lost in both *cep290ex25* morphants and *MZcep290^−/−^* mutants (Fig. 1E,F).

**Fig. 2.**
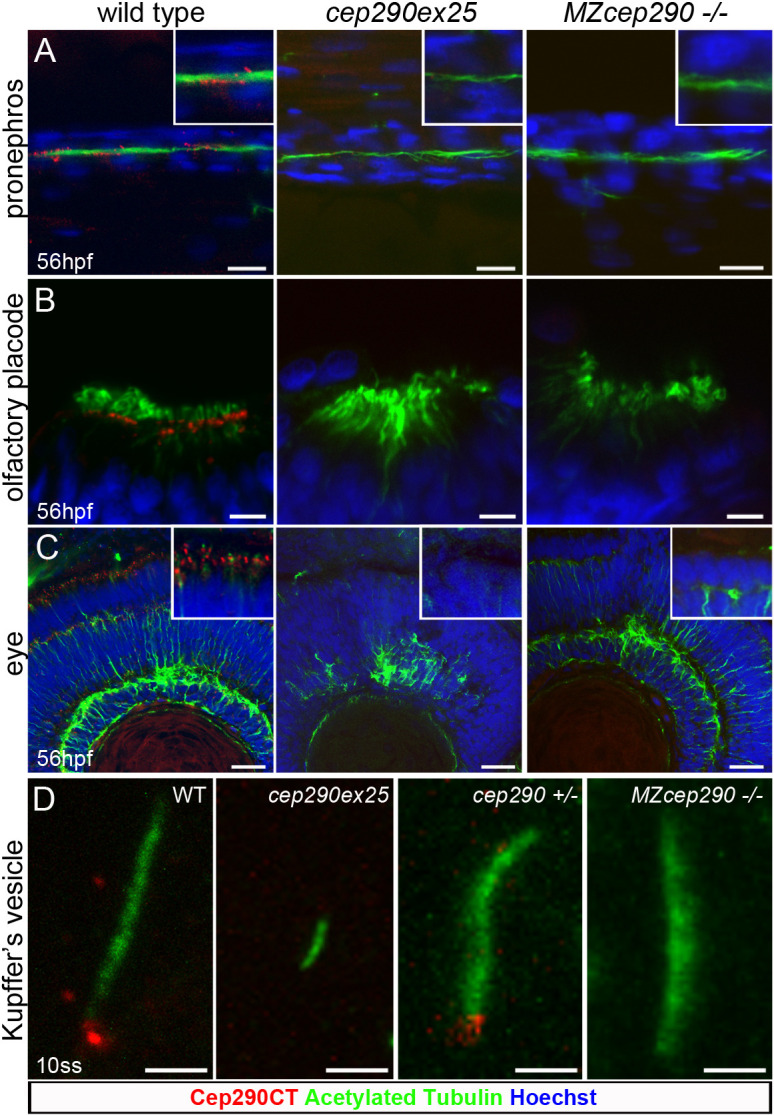
**Functional loss of Cep290 in zebrafish morphants and mutant embryos.** (A-C) Cep290CT antibody staining was lost in pronephric tubule multicilia bundles (A), at the base of nose multicilia (B) and in photoreceptor, outer and inner plexiform retina layers (C) upon injection of the *cep290ex25* morpholino or in *MZcep290^−/−^* mutants. Loss of Cep290 expression caused an alteration of retina lamination. No effect was observed on pronephros and olfactory placode cilia structure. (D) Kupffer's vesicle cilia were significantly shorter in 10ss *cep290ex25* morphant embryos compared to stage-matched embryos injected with control morpholino. By contrast, 10ss *MZcep290* mutant embryos did not show shorter Kupffer's vesicle cilia compared with heterozygous sibling embryos. Images are representative of >8 embryos/biological replicates. Experiments were replicated a minimum of three times. Scale bars: 10 µm (A); 5 µm (B); 20 µm (C); 2 µm (D).

To further assess the impact of knockdown versus genetic mutation in *cep290*, we imaged whole-mount zebrafish embryos using confocal immunofluorescence. Both C-terminal and N-terminal anti-Cep290 antibodies localized Cep290 to the base of the cilia in all tissues examined (Fig. S2). Staining of basal body domains in Kupffer's vesicle was eliminated by preincubation of the antibodies with free antigen (Fig. S2A,B), demonstrating the specificity of immunoreactivity. Consistent with previous reports (Chang et al., 2006; Kim et al., 2008; Lopes et al., 2011; Rachel et al., 2012, 2015; Sang et al., 2011; Sayer et al., 2006; Stowe et al., 2012; Valente et al., 2006), confocal imaging revealed that zebrafish Cep290 protein localization was cell-type specific, showing a transition zone distribution in olfactory placode, photoreceptor and pronephric cilia, and a pericentriolar satellite localization in Kupffer's vesicle, photoreceptor and spinal canal cilia (Fig. S2C-J). Both maternal zygote mutation and knockdown of *cep290* caused a complete loss of Cep290 protein immunoreactivity in the cilia basal body/transition zone (Fig. 2). Cilia in the pronephros (multiciliated cells), olfactory placode motile cilia, photoreceptors and Kupffer's vesicle cilia were devoid of Cep290 expression, confirming protein loss in both conditions. Despite the complete absence of Cep290 immunoreactivity in both morpholino and genetic deficiencies, confocal analysis revealed differences in phenotypes depending on the type of *cep290* deficiency. Consistent with previous reports, no cilia defects were observed in pronephric or olfactory placode cilia in *cep290ex25* morphants and *MZcep290^-/-^* mutants (Sayer et al., 2006; R. Bachmann-Gagescu personal communication; Fig. 2A,B), whereas loss of *cep290* expression caused various degrees of defects in retinal lamination in *cep290ex25* morphants and *MZcep290^-/-^* mutants, as suggested by a disorganization of the outer and inner plexiform retinal layers (Fig. 2C). However, quantification of Kupffer's vesicle cilia length showed that *cep290ex25* morphant embryos present significantly shorter Kupffer's vesicle cilia than *MZcep290* mutant cilia (Fig. 2D; Fig. S3; 1.76±0.55 µm cep290ex25MO versus 5.89±1.23 µm control; 6.28±1.14 µm *MZcep290^−/−^* versus 5.7±1.15 µm *cep290^+/−^*; mean±s.d.).

**Fig. 3.**
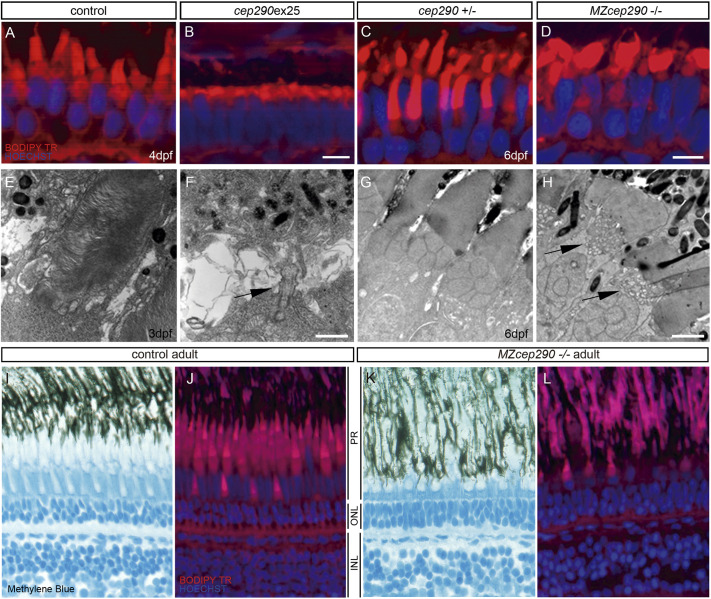
**Cep290 morphants and mutants exhibit photoreceptor outer segment length defects.** (A-D) BodipyTR (red) staining showed a reduction of photoreceptor outer segment length of 4-dpf *cep290ex25* morphants compared with age-matched control morpholino-injected embryos (A,B). *MZcep290* mutants also showed a disorganization of photoreceptor outer segment structure at 6 dpf (C,D). (E,F) Electron micrographs of 3-dpf wild-type and *cep290ex25* morphant photoreceptors. Although outer segments were found in wild-type retinas at this stage, very few outer segments were observed in *cep290ex25* morphants. In some cases, basal bodies and short axonemes could be seen projecting from the cell surface (arrow in F). (G,H) Electron micrographs of 6-dpf heterozygous sibling and *MZcep290* mutant photoreceptors showed an abnormal accumulation of cytoplasmic vesicles at the base of the connecting cilium (arrows in H). (I-L) Photoreceptor structure alteration persists until adulthood. Methylene Blue (I,K) and BodipyTR (J,L) staining showed an alteration of photoreceptor outer segment structure and retina lamination in *MZcep290^−/−^* adult (1 year old) retinas. Images are representative of >8 embryos/biological replicates. INL, inner nuclear layer; ONL, outer nuclear layer; PR, photoreceptor. Experiments were replicated a minimum of three times. Scale bars: 5 µm (A-D); 50 nm (E-H).

Further analysis of photoreceptor structure revealed significant differences in phenotype severity between morpholino and genetic *MZcep290* deficiencies. BodipyTR staining of 6 dpf retinal sections revealed a severe reduction of photoreceptor outer segment length in *cep290ex25* morphants relative to controls (Fig. 3A,B; Murga-Zamalloa et al., 2011), whereas *MZcep290^-/-^* photoreceptors exhibited only mild disorganization of outer segment morphology without a significant change in outer segment length (Fig. 3C,D). Similarly, electron microscopy revealed a near absence of photoreceptor outer segments in *cep290ex25* morphants, with evidence of only residual basal bodies and short axonemes (Fig. 3E,F), whereas *MZcep290^−/−^* mutant outer segments were clearly present (Fig. 3G,H). Significantly, *MZcep290^-/-^* mutant photoreceptors showed an abnormal accumulation of cytoplasmic vesicles at the base of the connecting cilium of the photoreceptor (Fig. 3H), suggesting a defect or partial blockage in cilia membrane trafficking in the mutant. Despite altered photoreceptor structure in *MZcep290^−/−^* larvae, no impairment of visual function could be detected by electroretinogram (ERG) analysis (data not shown). Altered photoreceptor outer segment structure and retina lamination persisted in *MZcep290^−/−^* adult retinas (Fig. 3I-L) (Chang et al., 2006; Lessieur et al., 2019; Sayer et al., 2006; Shimada et al., 2017). Taken together, the data indicate that *cep290* loss-of-function phenotypes of varying severity are only observed in tissues and cells in which Cep290 is localized to pericentriolar satellites in which Cep290 is known to play a role in cilia membrane transport. The data also show that acute inhibition of *cep290* expression produces more severe cilia defects than a long-term genetic loss of function, consistent with previous reports (Baye et al., 2011; Lessieur et al., 2019; Murga-Zamalloa et al., 2011; Sayer et al., 2006; Stawicki et al., 2016).

### Cep290 mutant expressivity is not due to exon skipping or nonsense-mediated decay mechanisms

**Fig. 4.**
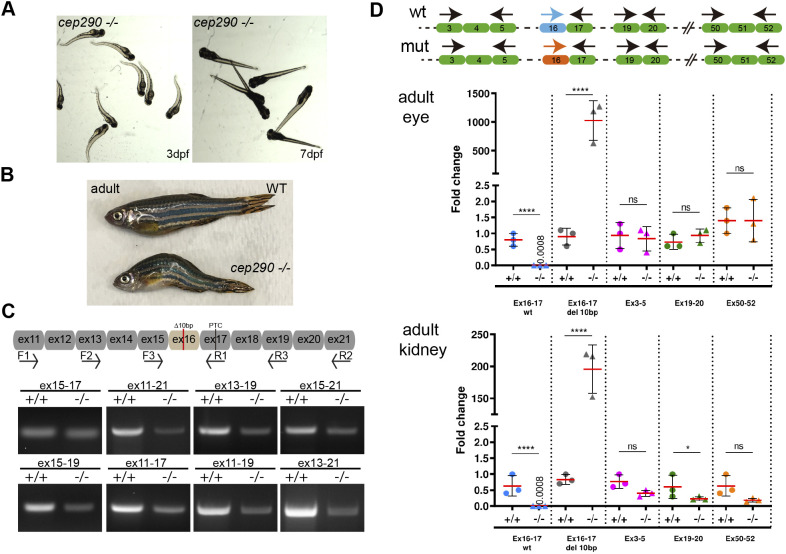
***MZcep290^−/−^* mutants present milder phenotypes that recover after 4-5 dpf.** (A) Clutch of *cep290* maternal zygotic *cep290* mutants. Body shape curvature seen at 3 dpf was lost at 7 dpf (*n*=100). (B) Maternal zygotic cep290 mutants reach adulthood and can present scoliosis (15-40% of adult fish; *n*=31). (C) RT-PCR from *MZcep290^−/−^* and wild-type embryos. No exon skipping around exon 16 was detected in mutant embryos. (D) RT-qPCR from adult eye and kidney samples (*n*=3). Specific wild-type (WT) and mutant primers for the targeted exon 16 were used to quantify wild-type and mutated cep290 mRNA. As expected, wild-type primers did not generate an RT-PCR product with mutant mRNA samples relative to wild-type mRNA, whereas mutant primers generated significant product with mutant mRNA samples relative to wild-type mRNA samples. Primers for ex3-ex5, ex19-ex20 and ex50-52 were used to quantify nonsense-mediated decay. No changes in cep290 mRNA levels were detected in *MZcep290^−/−^* mutant samples compared with controls. *n*=3 biological replicates. Data are mean±s.d. Experiments were replicated at a minimum three times. *P<0.05; ****P<0.0001; ns, not significant (unpaired *t*-test).

Mild phenotypes in *cep290* and other zebrafish genetic mutants versus acute knockdowns have been puzzling (Chang et al., 2006; Drivas et al., 2015; Lessieur et al., 2019; Rachel et al., 2015; Sayer et al., 2006). We noted that although 3 dpf *MZcep290^−/−^* mutants initially show an axis curvature phenotype similar to the morpholino knockdown phenotype, axis curvature recovered to wild-type axis shape by 7 dpf (Fig. 4A), at which stage mutants are indistinguishable from wild type. Adult *MZcep290^−/−^* mutants showed various degrees of fertility and spinal scoliosis (Fig. 4B), suggesting the axis phenotype was transiently and partially suppressed. To further explore the possibility of phenotype suppression in *MZcep290*^−/−^ mutants, we assayed for nonsense-associated altered splicing of mutated exons, as has been reported to account for varying expressivity in *CEP290* patients (Barny et al., 2018; Littink et al., 2010; Ramsbottom et al., 2020). RT-PCR analysis of mutant and wild-type *cep290* mRNA exon structure, using primers designed for ten exons flanking the mutated exon 16 (Fig. 4C), did not detect changes in amplicon sizes between wild type and mutants, suggesting that exon skipping does not account for differing degrees of *cep290* deficiency phenotype severity. Alternatively, as the mutation in exon 16 of *cep290* is a 10-bp deletion it is predicted to produce a frameshift and a premature termination codon that may induce nonsense-mediated mRNA decay. Therefore, we compared the levels of mutated and wild-type *cep290* mRNA by qRT-PCR (Fig. 4D). As expected, primers specific to the wild-type exon 16 sequence did not amplify mRNA from mutants and conversely primers specific to the deleted exon 16 sequence (Ex16-17 del10pb) selectively amplified *MZcep290* mutant mRNA (Fig. 4D). Importantly, in adult eye and kidney RNA, samples amplified using primers upstream (ex3-ex5) and downstream of the mutated exon (ex19-ex20 or ex50-ex52) did not detect significant differences in *cep290* mRNA levels between maternal zygote mutant and control animals, although there was a tendency for reduction in kidney samples. The results show that the presence of a *de novo* premature termination codon in the *cep290* mutant mRNA did not activate nonsense-mediated decay, as has been reported in other CRISPR/Cas9 mutant lines (Anderson et al., 2017). Despite the persistence of the mRNA, we did not detect the 290-kDa protein with the N-terminal and C-terminal Cep290 antibodies in maternal-zygotic mutant samples (Fig. 1F), indicating no full-length Cep290 protein was produced.

### Genetic mutation in zebrafish cep290 induces upregulation of genes encoding Cep290 interactors involved in cilia membrane transport

**Fig. 5.**
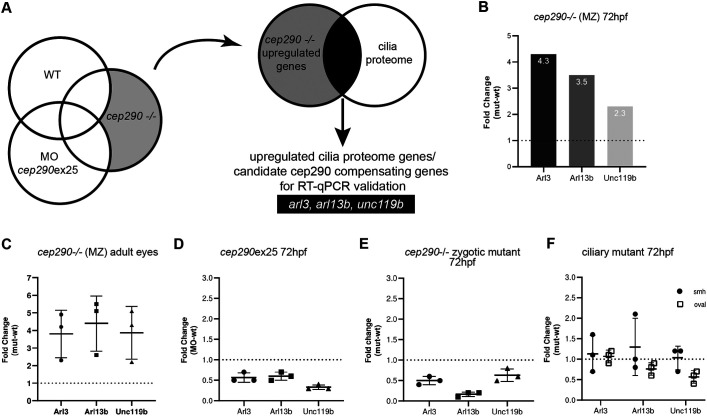
***arl3*, *arl13b* and *unc119b* are upregulated in *MZcep290* mutants.** (A) RNAseq workflow for the identification of candidate cep290 compensating genes. RNAseq gene expression analysis was performed with 3-dpf *cep290ex25* morphants, *cep290* mutants and sibling wild-type (WT) embryos (NCBI GEO accession GSE175491), with three biological replicates. Upregulated genes in each group were identified, and genes specifically upregulated in *MZcep290^−/−^* mutants were selected for further analysis. *MZcep290* upregulated genes were compared to a cilia proteome database to identify candidate cep290 compensating cilia-related genes. (B) RNAseq results showing upregulation of cilia-related genes *arl3*, *arl13b* and *unc119b* in 72-hpf embryos. (C) RT-qPCR quantification of *arl3*, *arl13b* and *unc119b* from *MZcep290* mutant adult eye samples. *n*=3 biological replicates per condition; technical triplicates. (D) The upregulation of *arl3*, *arl13b* and *unc119b* was not detected at 3 dpf in *cep290ex25* morphants embryos. *n*=20 embryos pooled per condition; technical triplicates. (E) The upregulation of *arl3*, *arl13b* and *unc119b* also was not detected at 3 dpf in *cep290* zygotic mutants embryos. *n*=20 embryos pooled per condition; technical triplicates. (F) The ciliary mutants *smh* (*ccdc103*) and *oval* (*ift88*) did not show upregulation of *arl3*, *arl13b* and *unc119b* at 3 dpf. *n*=20 embryos pooled per condition; technical triplicates. All RT-qPCR data are expressed as fold change (2^−ΔΔCt^) compared with age-matched control embryos. Data are mean±s.d.

To explore other possible genetic compensation mechanisms in the *cep290* mutant line, we performed RNAseq analysis of the *cep290* morphants, maternal zygote mutants and wild-type siblings, and screened for genes specifically upregulated in the mutant that may compensate for Cep290 deficiency (NCBI GEO accession number GSE175491; Fig. 5A). Comparison of wild-type, *cep290* morphant and *MZcep290* mutant transcriptomes revealed a set 1953 genes upregulated >2-fold in *MZcep290^−^*^/^*^−^* mutants versus wild-type larvae (Tables S1-S3). Functional annotation of this gene set using DAVID (Huang et al., 2009) revealed enrichment for RNA binding, endoplasmic reticulum membrane and small GTPase proteins (Table S4). We further sorted this set for genes encoding cilia or centrosome proteins (CiliaCarta; van Dam et al., 2019), genes enriched in zebrafish multiciliated cells (Tang et al., 2017), genes present in cilia proteome studies (Ishikawa et al., 2012; Li et al., 2004; Mayer et al., 2009; Nogales-Cadenas et al., 2009; Pazour et al., 2005) or genes associated with ciliopathies (DAVID annotation; Huang et al., 2009), to ascertain candidate suppressors of the *cep290* mutant deficiency (Table S5). Of 22 cilia-related candidate genes upregulated in *cep290* mutants, the cilia-associated genes *arl3*, *arl13b* and *unc119b* showed a 4.3-, 3.5- or 2.3-fold induction in *MZcep290^−/−^* embryos, respectively (Fig. 5B), and were validated by RT-qPCR. All three of these genes participate in the targeting and delivery of lipidated proteins to cilia membranes. Unc119 (Unc-119 lipid binding chaperone B) is a myristoyl-binding protein that acts as a cargo adapter for lipidated protein transport, Arl3 (ADP-ribosylation factor-like 3) is a small GTPase involved in the targeting and releasing of lipidated cargo proteins from their carriers at the cilium (Powell et al., 2019; Zhou et al., 2006), and Arl13b (ADP ribosylation factor-like GTPase 13b) is the guanine nucleotide exchange factor that activates Arl3 (Duldulao et al., 2009; Gotthardt et al., 2015; Nozaki et al., 2017). We confirmed that upregulation of *arl3*, *arl13b* and *unc119b* assayed in larvae also occurred in *MZcep290^−/−^* adult eye samples (Fig. 5C) but not in *cep290ex25* morphants or *cep290^−/−^* zygotic mutant embryos, which displayed prominent axis curvature (Fig. 5D,E). To determine whether upregulation of *arl3*, *arl13b* and *unc119b* was specific to *cep290* deficiency, we analyzed the zebrafish cilia mutant lines *oval* (*ift88*) and *schmalhans* (*ccdc103*) (Panizzi et al., 2012; Tsujikawa and Malicki, 2004). No upregulation of these genes was detected (Fig. 5F), suggesting that *arl3*, *arl13b* and *unc119b* upregulation was a specific consequence of the *MZcep290* gene mutation. Our transcriptomic analysis suggests that upregulation of GTPases and cargo adaptor proteins involved in the delivery of cilia membrane proteins may compensate for the *MZcep290* deficiency.

### UNC119B is upregulated in Joubert patient-derived cells

**Fig. 6.**
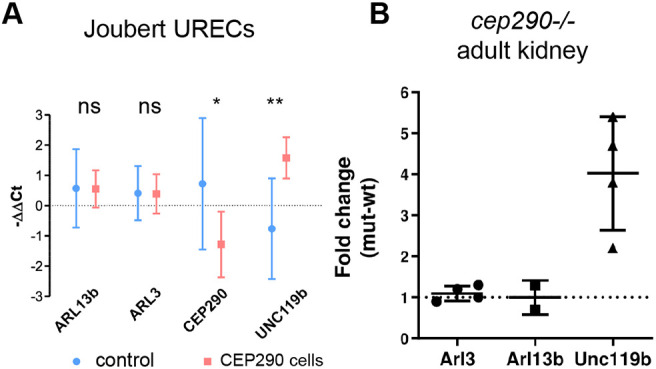
**Joubert patient-derived cells also show upregulation of UNC119b.** (A) RT-qPCR analysis from hURECs from JBTS *CEP290* patients showed upregulation of *UNC119B* expression compared with control patient samples. No change in *ARL3* and *ARL13B* gene expression level was observed. Values (dots) represent arithmetic means of −ΔΔCt values between experimental replicates (three controls, four CEP290 patients). Bars represent 95% c.i. *P<0.05; **P<0.01; ns, not significant (unpaired *t*-test, two-tailed). (B) In zebrafish *MZc*ep290^−/−^ adult kidney samples also only *unc119b* induction was observed. *n*=3 biological replicates per condition, technical triplicates. RT-qPCR data are expressed as fold change (2^−ΔΔCt^) compared with age-matched control samples. Data are mean±s.d.

JBTS in humans is a genetically heterogeneous autosomal-recessive multisystem ciliopathy, in which mutations in *CEP290* are a common underlying cause of cerebello-retinal-renal phenotypes (Sayer et al., 2006; Valente et al., 2006). We analyzed urine-derived renal epithelial cells (URECs) from JBTS patients by RT-qPCR for the expression of *ARL3*, *ARL13B* and *UNC119B* to assay for genetic compensation in humans. Significantly, we detected a 4-fold induction of *UNC119B* compared with control patient UREC samples (Fig. 6A). No change in ARL3 or ARL13B expression was observed. Similarly, in adult zebrafish kidney, qRT-PCR analysis showed a 4-fold upregulation of *unc119b* but not *arl3* or *arl13b* expression (Fig. 6B). Upregulation of genes encoding cilia membrane protein transport factors appears to be a conserved mechanism associated with *cep290* mutation.

### Expression of arl3, arl13b and unc119b rescues cep290ex25 morphant axoneme defects

**Fig. 7.**
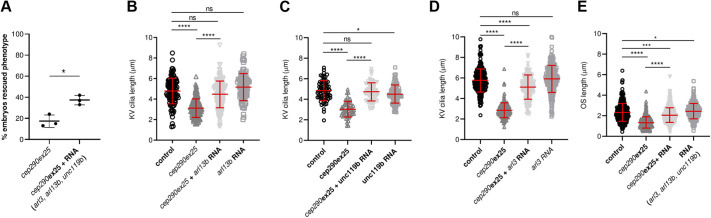
**Upregulation of small GTPases/chaperones that mediate cilia lipidated protein delivery rescue cep290ex25 morphant axoneme defects.** (A) Overexpression of cilia GTPases/chaperone (*arl3*, *arl13b* and *unc119b*) in *cep290ex25* morphant embryos partially rescued the curly axis cilia defect related phenotype. (B) *arl13b* overexpression was sufficient to rescue Kupffer's vesicle (KV) cilia length defects observed in stage-matched *cep290ex25* morphant embryos. (C) Kupffer's vesicle cilia length was also rescued by overexpression of *unc119b* mRNA. (D) Injection of *arl3* mRNA only partially rescued Kupffer's vesicle cilia length in *cep290ex25* morphant embryos. (E) The injection of *arl3*, *arl13b* and *unc119b* mRNAs also was sufficient to rescue the photoreceptor outer segment length defect on *cep290ex25* morphants. *P<0.01; ***P<0.001; ****P<0.0001; ns, not significant (Dunnett's or Tukey's test). *y*-axis values represent the average length of >70 Kupffer's vesicle cilia from 3-6 embryos per condition or of >200 OS from four embryos per condition. Data are mean±s.d.

To functionally test whether upregulation of cilia membrane transport factors could compensate for *cep290* deficiency, we assayed the effect of *arl3*, *arl13b* and *unc119b* overexpression on *cep290* phenotypes in acute knockdowns in which we saw strong ciliopathy phenotypes and no compensatory gene expression (Fig. 5D). We injected zebrafish *arl3*, *arl13b* or *unc119b* synthetic mRNAs in combination with the *cep290ex25* morpholino in 1-2 cell embryos and analyzed the outcome on three measurable phenotypes: body curvature, Kupffer's vesicle cilia length and photoreceptor outer segment length (Fig. 7). The injection of *cep290ex25* morpholino alone produced 86±1.4% (mean±s.d.) of embryos with curly phenotype and 14% with straight body shape, perhaps due to failure on morpholino delivery in those embryos (Fig. 7A). Interestingly, upon the injection of the three RNAs in *cep290* morphants, we observed an increase in 37.5±6.3% in embryos with a straight body shape, suggesting that expression of *arl3*, *arl13b* or *unc119b* is able to compensate for acute *cep290* loss of function.

Acute knockdown of *cep290* using the *cep290ex25* morpholino caused a shortening of Kupffer's vesicle cilia, whereas *MZcep290^-/-^* mutation did not (Fig. 2D; Fig. S3). We therefore tested whether *arl3*, *alr13b* or *unc119b* mRNA expression was able to rescue Kupffer's vesicle cilia defects in *cep290* morphant embryos. Injection of each gene individually or in combination rescued cilia length defects in the Kupffer's vesicle caused by *cep290* morpholino knockdown (Fig. 7B-D; Fig. S4A-C). Injection of *arl13b* and *unc119b* RNA in morphant embryos completely rescued Kupffer's vesicle cilia, reaching control cilia length (4.47±1.3 µm Arl13bRNA plus cep290ex25MO versus 4.79±1.3 µm control; 4.73±0.9 µm unc119bRNA plus cep290ex25MO versus 4.83±1.0 µm control; mean±s.d.; Fig. 7B,C). Expression of *arl3* RNA also rescued the length of the cilia but only partially (5.11±1.2 µm Arl3RNA plus cep290ex25MO versus 5.79±1.2 µm control; *P*≤0.0001; Fig. 7D), perhaps due to reduced stability of the injected mRNA (Fig. S4D). Injection of mRNA encoding Rab8a, a Cep290 interactor, did not rescue Kupffer's vesicle cilia length (Fig. S5A), suggesting that overactivation of cilia initiation and apical transport alone was not sufficient to overcome *cep290* deficiency. On the other hand, overexpressing the cilia-targeted membrane protein Sstr3-GFP was sufficient to rescue Kupffer's vesicle cilia length (Fig. S5B), suggesting that ciliated cells can respond to increased cilia transport demand and overcome a deficiency in individual components of the cilia membrane protein transport pathway. Injection of *arl3*, *arl13b* and *unc119b* mRNAs alone, or in combination with *cep290ex25* morpholino, also induced a partial rescue of photoreceptor outer segment length defects in *MZcep290* morphant embryos (2.07±0.72 µm RNA plus cep290ex25MO versus 2.32±0.88 µm control; mean±s.d.; Fig. 7E; Fig. S6), indicating compensation occurred in multiple tissues affected by *cep290* mutation. Taken together, our results indicate that a genetic deficiency in *cep290* activates expression of additional related components of the cilia membrane protein transport pathway, which allows for ciliogenesis and relatively normal cilia function in *cep290* mutants. This phenomenon constitutes an additional mechanism contributing to the observed range of expressivity in *cep290* deficiencies.

## DISCUSSION

*CEP290* mutations have been associated with several ciliopathies that vary widely in their severity and clinical manifestations (Reiter and Leroux, 2017). CEP290 deficiency in humans can be as mild as isolated blindness (Leber's congenital amaurosis) or as severe as perinatal lethality (Meckel–Gruber syndrome) (Coppieters et al., 2010). Given the range of defects seen in human individuals with CEP290 variants, zebrafish mutant phenotypes are surprisingly mild and limited to photoreceptor malformation, subtle vision defects and scoliosis in a subset of mutants (Lessieur et al., 2019). Understanding the variable expressivity of *CEP290* mutations has been puzzling, and several explanations have been proposed to explain the poor genotype-phenotype correlations observed in patients and animal models (Coppieters et al., 2010; Drivas et al., 2015). In the present study, we show that genetic compensation by upregulation of GTPase regulators of lipidated cilia protein delivery constitute an additional mechanism contributing to the variable expressivity of *cep290* deficiencies. We also provide further evidence that Cep290 subcellular localization is cell type specific either in the cilia transition zone or pericentriolar satellites. Further, Cep290 localization reflects its function as only cells in which Cep290 is localized specifically to pericentriolar satellites show mutant and knockdown ciliopathy phenotypes.

### Phenotypic variation and compensation of Cep290 deficiency

Our result that expressivity of Cep290 deficiencies is dependent on the type of genetic ablation (mutation or knockdown) is consistent with a recent report on the phenotype of the *cep290* TILLING mutant *cep290^ fh297/fh297^.* This mutation introduces a premature stop codon at exon 29 and displays mild phenotypes, such as sigmoidal tail curvature and progressive cone degeneration (Lessieur et al., 2019). These results indicate that altering *cep290* gene expression at a genomic level in zebrafish induces mild phenotypes independently of the gene region that is affected. Compared to strong ciliopathy phenotypes in acute *cep290* knockdown zebrafish (Baye et al., 2011; Murga-Zamalloa et al., 2011; Sayer et al., 2006), the results indicate that an adaptive compensation mechanism may suppress a genetic mutant phenotype.

Genetic compensation, including transcriptional adaptation in genetic mutants, has been proposed to be mediated by the upregulation of paralogous genes, modifier genes or genes that function in a similar biological process or pathway (El-Brolosy et al., 2019; Nadeau, 2001; Sztal et al., 2018). In transcriptional adaptation, genes paralogous to the mutant gene are upregulated by a mechanism involving nonsense-mediated decay associated with a premature termination codon mutation, generation of short RNA fragments and derepression of paralog transcription to compensate for loss of the mutant gene function (Sztal and Stainier, 2020). However, in some mutants, expression of compensatory genes is induced without degradation of the mutant mRNA (Buglo et al., 2020; Ma et al., 2019). Currently, no paralog of *cep290* has been reported in the current assembly of the zebrafish genome, making it unlikely that this type of mechanism can account for the mild phenotype of *cep290* mutants versus morphants. Additionally, we saw no evidence for nonsense-mediated mRNA decay in *cep290* mutants. Instead, we observed that small GTPases associated with cilia membrane protein delivery were upregulated in *cep290* mutants that could compensate for the loss of Cep290 function in acute knockdowns.

Currently, the mechanism for this compensation is unknown but it is significant that the compensating genes function in a subcellular pathway central to Cep290 activity (Craige et al., 2010; Drivas et al., 2013; McEwen et al., 2007; Tsang et al., 2008). Arl3 and Arl13b are small GTPases that interact with the adaptor protein Unc119b to mediate cilia lipidated membrane protein transport (Alkanderi et al., 2018; Duldulao et al., 2009; Gotthardt et al., 2015; Nozaki et al., 2017; Powell et al., 2019). Unc119b also interacts with the NPHP-MKS protein complex, which is involved in ciliary gate function at the cilia transition zone and interacts with CEP290 through NPHP5 (Sang et al., 2011; Schäfer et al., 2008; Williams et al., 2011; Wright et al., 2011). It is noteworthy that Lessieur et al. (2019) recently analyzed genetic interactions of *arl13b* with *cep290* in zebrafish. They showed that removing one allele of *cep290* significantly enhanced the defects in both visual contrast sensitivity and spatial frequency of *arl13b^−/−^* mutants, which show progressive photoreceptor degeneration (Song et al., 2016), arguing that *cep290* is a modifier gene of *arl13b*. Although the loss of one allele of *arl13b* in *cep290fh297/fh297* mutants did not accelerate photoreceptor degeneration, these results nonetheless imply that *arl13b* and *cep290* are functionally interrelated. Further experiments inducing full loss of *arl13b* or *unc119b* function in a *cep290*-deficient larvae may show more severe ciliopathy phenotypes. Compensation of ciliopathy by upregulation of *arl3*, *arl13b and unc119b* appears to be specifically induced by mutation in the *cep290* gene as we did not observe upregulation of these genes in the cilia motility mutant *smh* (*ccdc103*) or the IFT mutant *oval* (*ift88*). This type of genetic compensation has also recently been shown in zebrafish mitochondrial gene mutants, in which stable mutation of the mitochondrial carrier *slc25a46* gene induces upregulation of the functionally related calcium binding protein annexin 6A, resulting in the absence of phenotypes in *slc25a46* mutants (Buglo et al., 2020). Significantly, mutation in the zebrafish *giantin* gene, which encodes a golgin protein involved in golgi structure and is required for ciliogenesis (Bergen et al., 2017; Stevenson et al., 2017), has been shown to be compensated by upregulation of the regulator of the calcineurin 2 (RCAN2) gene. RCAN2 was identified as the gene most highly upregulated by *giantin* genetic knockout, and was shown by knockdown to suppress ciliopathy phenotypes in *giantin* mutants (Stevenson et al., 2018).

Interestingly, our results indicate that the genetic compensation in *cep290* mutants may also be tissue specific as we observed differences in expression of compensatory genes between eye and kidney samples. The adult retina of *cep290* mutants shows mild disorganization of retinal layers and photoreceptor outer segment structures, along with upregulation of *arl3*, *arl13b* and *unc119b*, suggesting attenuation of retina defects but not complete suppression. This is consistent with our rescue experiments in *cep290* morphant larvae in which we were only able to partially suppress retinal phenotypes by ectopic *arl3*, *arl13b* and *unc119b* expression. In contrast, adult kidney *cep290* mutant samples only showed upregulation of *unc119b*, indicating that *unc119b* may have a more central function in kidney cells. Importantly, we also observed this result in kidney samples derived from human JBTS patients, suggesting that this mechanism is conserved in humans and may contribute to the pleiotropy of *CEP290* mutations seen in humans.

### *Cep290* mutant phenotype severity correlates with cell type-specific subcellular protein localization

Different levels of compensatory gene expression could also be related to cell type-specific Cep290 protein localization and function (Rachel et al., 2015). It is noteworthy that *cep290* deficiencies, either by knockdown or gene mutation, most strongly affect cells in which Cep290 is localized to pericentriolar satellites as opposed to the cilia transition zone. In photoreceptors, outer segment membrane discs undergo rapid turnover, necessitating elevated levels of protein and membrane transport through the connecting cilium to maintain cilia length (Bachmann-Gagescu and Neuhauss, 2019). Indeed, we observed that dysfunction in this trafficking system led to a detectable accumulation of membrane vesicles in *cep290* mutant photoreceptors. Demand for cilia membrane and protein delivery may be less in kidney cilia that do not turn over as rapidly, and hence may be less affected by *cep290* mutation and exhibit reduced compensatory gene expression. We also acknowledge that compensation may be *cep290* allele or gene dosage specific as we did not observe gene upregulation in *cep290* mutant heterozygotes.

### Cilia membrane protein delivery and regulation of axoneme length

Our results showing ectopic expression of either *arl3*, *arl13b* or *unc119b* was able to rescue *cep290* knockdown Kupffer's vesicle cilia length support recent studies implicating lipidated cilia membrane protein transport in regulating axoneme length (Broekhuis et al., 2013; Fisher et al., 2020; Jensen and Leroux, 2017; Li et al., 2012; Prevo et al., 2017). Arl13b acts as a positive regulator of ciliary length by inducing ciliary membrane protrusion and extension of axonemal microtubules, possibly via a direct interaction with tubulin G-domains (Lu et al., 2015; Revenkova et al., 2018). In contrast, Arl3 loss of function does not directly affect cilia length (Alkanderi et al., 2018; Hanke-Gogokhia et al., 2016; Schrick et al., 2006) but rather may affect ciliogenesis through its functional interaction with Arl13b and the solubilization factors Unc119b and Pde6d (Fansa et al., 2016; Gotthardt et al., 2015; Ismail et al., 2012, 2011; Jaiswal et al., 2016; Roy and Marin, 2019; Thomas et al., 2014; Wright et al., 2011; Zhang et al., 2016). In our studies, ectopic expression of either *arl3*, *arl13b* or *unc119b* mRNA alone was able to rescue *cep290* knockdown Kupffer's vesicle cilia length defects, providing evidence that the level of each gene may be rate limiting for ciliogenesis. Further, we showed that overexpression of an unrelated cilia transmembrane protein (Sstr3-GFP) rescued Kupffer's vesicle cilia length in *cep290* knockdown embryos, suggesting that driving cilia membrane delivery per se can be sufficient to overcome deficiencies in normal cilia length. Our results also suggest that not all ciliopathy genes are absolutely essential for ciliogenesis, and that for the relatively large *CEP290* gene, expressing small GTPases in affected tissues may be an alternative and more feasible route to gene therapy for human *CEP290* mutations.

In summary, our work shows that *cep290* gene mutation can trigger the expression of genes related to cilia membrane protein transport, allowing for ciliogenesis and relatively normal cilia function in *cep290* mutants. Although it remains to be determined how this mechanism is triggered and what the role of genetic background may be in this process, our findings constitute a new genetic compensation mechanism in ciliopathy that will further our understanding of genotype-phenotype discrepancies in *cep290* mutations.

## MATERIALS AND METHODS

### Zebrafish husbandry

Zebrafish (TuAB; *Danio rerio*) of both male and female sexes were used in the experiments. Animals were maintained and bred according to standard procedures (Westerfield, 2000), and experiments were performed in accordance with approved animal care guidelines under Massachusetts General Hospital and Mount Desert Island Biological Laboratory Institutional Animal Care and Use Committee protocols. Embryos analyzed in early somitogenesis were maintained at room temperature after fertilization; for studies of the adult retina, 1-year-old animals were used. Embryos analyzed later during development were maintained at 28°C. For immunofluorescence experiments, embryos were treated with 1-phenyl 2-thiourea after 24 hours post-fertilization (hpf) to reduce pigmentation.

### Morpholino knockdowns

Three antisense morpholinos were designed against zebrafish cep290, including two splice donor morpholinos and one translation blocking (ATG) morpholino. All morpholinos were acquired from Gene Tools: cep290ex25dmo, TTGATGTGTACCAGTTGTGCTGATG; cep290ex42dmo, AAAGTTGCACCTACAGAGGGTCTGG; and cep290ATGmo, GCCGCAGGCATTCTTCAGGTCAGCT. Control injections were performed using a mismatched morpholino: ctrlMO, CGTCCATTGTGTAAAAGTGTAACCA. Morpholinos were diluted to a final concentration of 0.33-0.76 ng/nl in a solution of 100 mM KCl, 10 mM HEPES and 0.1% phenol red. Volumes of 4.6 nl were injected into the yolk of one- to four-cell stage embryos using a Nanoliter2000 microinjector (World Precision Instruments). Molecular defects caused by splice-donor-targeted morpholino were assessed by reverse-transcription PCR on total RNA extracted from single morphant embryos. For this analysis, nested primers were designed to bind in exons flanking the morpholino target site. For primer sequences, see Table S6.

### Generation of Cep290 mutant line by CRISPR/Cas9 mutagenesis

Cas9 mRNA (1.4 ng/embryo) and gRNAs (58 pg/embryo) (Oligo1, 5′-TAGGAAGGCCTTAGGGATCTGAC-3′; Oligo2 5′-AAACGTCAGATCCCTAAGGCCTT-3′) were injected into one-cell stage zebrafish embryos. gRNA was directed against exon 16 of zebrafish Cep290 mRNA (NM_001168267.1). gRNas were produced by cloning into pDR274 (Hwang et al., 2013) and then used as template for T7 RNA polymerase (Maxi Script Ambion) transcription. gRNAs were purified on Qiagen RNAeasy columns. Injected embryos were analyzed by sequencing, and heterozygote founders were established by outcrossing grown GO fish with TUAB wild-type animals and genotyping F1 offspring. For primer sequences for genotyping, see Table S6.

### Reverse-transcriptase PCR and quantitative RT-PCR

RNA from embryos or adult samples was extracted using an RNeasy Plus Universal kit (Qiagen). cDNA was synthesized using SuperScript III First-Strand Synthesis System (Invitrogen) with random primers. Quantitative PCR was performed on a StepOnePlus thermal cycler using Sybr Green reagents (Applied Biosystems). Quantitative RT-PCR results are expressed as fold change by ΔΔCT, and analyzed as described previously (Willems et al., 2008). For primer sequences, see Table S6.

### Zebrafish Cep290 antibodies

Cep290 antibodies were produced and characterized as described previously (Bergboer et al., 2018). Briefly, antibodies were produced at Rockland Immunochemicals Inc. by immunizing rabbits with a maltose binding protein-D. rerio Cep290 fusion protein (Cep290 amino acids 2220-2396, C-term) or a 6x His-tagged D. rerio Cep290 protein fragment (amino acids 1-200, N-Term).

### Western blotting

Zebrafish embryo extracts were prepared from pools of 30-50 48 hpf embryos. Yolk was manually removed, and samples were homogenized using micropestles (Eppendorf) in 50 μl of buffer containing 150 mM NaCl, 50 mM Tris-HCl (pH 7.5), 1% NP-40, 1 mM Na2VO3, 50 mM NAF, 1 mM phenylmethylsulfonyl fluoride, 0.5% deoxicolate sodium and proteases inhibitor. Homogenates were centrifuged for 5 min at 13,000 ***g***, and samples were subject to SDS-PAGE electrophoresis and transferred to PVDF (Millipore) membranes. The following antibodies were used: Cep290CT (1:1000), Cep290NT (1:1000) (Bergboer et al., 2018), mouse anti-α-tubulin B512 (T5168; 1:5000, Sigma-Aldrich) and peroxidase-conjugated goat anti-mouse IgG and goat anti-rabbit IgG secondary antibodies (115-035-003 and 111-035-003, 1:10,000, Jackson ImmunoResearch).

### Whole-mount immunofluorescence and confocal microscopy

Embryos were fixed in Dent's fixative (80% MeOH and 20% DMSO) at 4C overnight. After rehydrating through a methanol gradient, embryos were blocked in PBS plus 0.5% Tween 20 (PBST), 10% normal goat serum and 1% DMSO for 2 h at room temperature. Primary antibody incubation was performed overnight at 4C in blocking solution. Primary antibodies were removed by PBST washes prior to incubation with Alexa Fluor-conjugated secondary antibody (Invitrogen) overnight at 4°C in blocking solution. After removal of secondary antibodies, embryos were washed, blocked and stained with a second set of primary and secondary antibodies following the same method. Primary antibodies used included Cep290CT (1:100), anti-acetylated tubulin 6-11B-1 (T6793, 1:500, Sigma-Aldrich), anti-gamma tubulin GTU88 (T6557; 1:500, Sigma-Aldrich), anti-GFP (ab13970, 1:500, Abcam), anti-IFT88 (1:500, a gift from Brian Perkins, Cleveland Clinic Foundation, Cleveland, OH). Immunoreactivity was detected using Alexa 546-, Alexa 488- and Alexa 405-conjugated goat anti-rabbit IgG (A-11010, A-11008 and A-31556, 1:1000, Thermo Fisher Scientific), Alexa 546-, Alexa 488- and Alexa 405-conjugated goat anti-mouse IgG (A-11030, A-11001 and A-31553, 1:1000, Thermo Fisher Scientific), and Alexa 488-conjugated goat anti-chicken (A-11039; 1:1000, Thermo Fisher Scientific) antibodies. Nuclei were stained by Hoechst (H3570, 1:2000, Thermo Fisher Scientific). For imaging, embryos were equilibrated in mounting medium (53% benzyl alcohol, 45% glycerol and 2% N-propyl gallate) and two- or three-color *z* series were acquired using a Zeiss LSM5 Pascal confocal microscope with a 63× oil immersion lens and sequential laser excitation. Acquired images were deconvolved using Huygens Essential Software (Scientific Volume Imaging). For analysis of Kupffer's vesicle and spinal canal cilia length, embryos were stage-matched prior to fixation to avoid nonspecific developmental delay induced by morpholino or RNA injection. Quantification of cilia length was performed on maximum intensity *z* projections of confocal stacks created in ImageJ. Cilia axonemes stained with anti-acetylated tubulin were traced and measured using the freehand line tool.

### Electron microscopy

Zebrafish embryos were fixed and embedded for standard transmission electron microscopy according to previously described methods (Drummond et al., 1998). For analysis of Cep290 localization in connecting cilia of the photoreceptor by immunoEM, we used a previously described post-embedding staining technique on embryos preserved using high pressure freezing and low-temperature embedding in Lowicryl resin (Nixon et al., 2009). For this protocol, an 18 nm colloidal gold goat anti-rabbit IgG secondary antibody (111-215-144, 1:10, Jackson ImmunoResearch) was used for immunodetection.

### mRNA injections

Zebrafish Arl3, Arl13b and unc119b open reading frames were subcloned from pME18S-FL3 (Dharmacon clones ID, 7440598, 7400648 and 7400443) into plasmid pCS2+ using an NEBuilder HighFidelity DNA Assembly Cloning system (New England Biolabs). Primers sequences are listed in Table S6. Capped RNAs were transcribed from linearized plasmids using a mMessage mMachine SP6 kit (Ambion/Thermo Fisher Scientific). Transcripts were DNase treated prior to purification using an RNeasy Mini Kit (Qiagen). Capped mRNAs were diluted to 10.86 ng/μl in RNase-free water, and injected at 4.6 nl volumes into single-cell zebrafish embryos.

### Photoreceptor outer segment staining and length quantification

Dehydrated embryos or adult eyes were embedded in JB-4 plastic resin (Polysciences) and then sectioned (5 μm) using a LEICA RM 2165 rotary microtome. Sections, in which optic nerve was present, were stained with Hoechst (H3570, 1:2000, Thermo Fisher Scientific) and BODIPY TR Methyl Ester (C34556, 1:200, Thermo Fisher Scientific), and mounted using Permount (SP15-500; Thermo Fisher Scientific). Two-color *z* series were acquired using a Zeiss LSM5 Pascal confocal microscope via sequential laser excitation with 40× and 63× oil objectives. Images were deconvolved using Huygens Essential software and processed using Adobe Photoshop. Adult eye sections were stained with Methylene Blue/Azure II (Humphrey and Pittman, 1974) and examined using a Nikon E800 microscope equipped with a Spot Insight charged-couple device digital camera. Quantification of photoreceptor outer segment (OS) length was performed on maximum intensity *z* projections of confocal stacks created in ImageJ. OSs were traced and measured using the freehand line tool.

### Statistical analyses

Power calculations were performed based on a difference of means with a confidence level of 0.95, a power of 80% and variance calculated based on pilot experiments. For experiments that showed large variance (Kupffer's vesicle cilia length, OS length), minimum sample size was exceeded to increase confidence level and power. Normal distribution of data sets was tested using a Shapiro–Wilk test. Comparison of two groups with normal distribution was analyzed by unpaired *t*-test, and three or more groups by one-way ANOVA with posterior Tukey's test. Data that did not follow normal distribution were analyzed using non-parametric tests, a two-tailed Mann–Whitney test for two sets of data or a Kruskal–Wallis test to compare more than two sets. *P*<0.05 was considered to be significant. Embryos were chosen from the same clutch for controls and morpholino injection at random, and no exclusion criteria were applied. Embryos and larvae were used from the same clutch (siblings) where possible to minimize variance. For comparisons of phenotypes with *n*<5, individual data points were plotted. Analysis was performed using GraphPad Prism version 8.0 for Mac OS X (GraphPad Software, San Diego, CA, USA, www.graphpad.com).

### RNAseq analysis

RNA was extracted from pools of 30 embryos of each condition (cep290ex25MO, MZcep290^−/−^, wild type). Samples were processed and analyzed at the Massachusetts General Hospital NexGen Sequencing Core using the Illumina HiSeq platform. PolyA selection and NEBNext Ultra Directional RNA Library Prep Kit for Illumina was used for mRNA library construction. Samples were barcoded and multiplexed, and run in two lanes to ensure equivalent read depth in 50-bp single-end reads. Quality filtered reads were aligned to the zebrafish genome (Zv9) using STAR (Dobin et al., 2013), and gene expression counts were calculated using the Python package HTSeq (Anders et al., 2015). The R package ‘edgeR’ (Robinson et al., 2010) was used to analyze differential gene expression.

### Isolation of patient URECs

Following informed and written consent, human URECs (hURECs) were isolated from urine collected from two JBTS *CEP290* patients and two unrelated wild-type controls. One JBTS patient carried in homozygosis the common *CEP290* variant NM_025114.4; c.5668G>T; p.G1890X, whereas the second JBTS patient was compound het for the *CEP290* variants c.5668G>T; p.G1890X and c.2495_2512delInATCT; p.T832Nfs*12. hURECs were cultured as described previously (Molinari et al., 2020). Briefly, cells were isolated from urine samples through repeated centrifugation passages and kept for the first 96 h in Dulbecco's medium Eagle medium/high glucose and Ham's F12 nutrient mix (1:1), supplemented with 10% (v/v) fetal bovine serum (FBS), 140 U/ml penicillin, 140 μg/ml streptomycin, 3.5 μg/ml amphotericin B and REGM SingleQuot kit supplements (Lonza), at 37°C in a humidified atmosphere of 5% (v/v) CO_2_. After isolation for 96 h, medium was changed to Renal Epithelial Cell Growth Basal Medium (REBM, Lonza) supplemented with 0.5% FBS, 140 U/ml penicillin, 140 μg/ml streptomycin, 3.5 μg/ml amphotericin B and REGM SingleQuot kit supplements (Lonza). Before collection, cells were cultured in the same medium devoid of FBS for 48 h to induce ciliogenesis.

### RNA preparation and RT-PCR

Total RNA from URECs was isolated using an RNeasy mini kit (Qiagen) according to the manufacturer's instructions, and quantified using a NanoDrop 2000 spectrophotometer. RNA (1 μg) was reverse transcribed using an oligo-dT primer and SuperScript III Reverse Transcriptase (Thermo Fisher Scientific). The resulting cDNA was used pure in RT-PCR reactions and diluted 10-fold or 5-fold in nuclease-free water for qPCR experiments. PrimeTime qPCR probe-based assay (Integrated DNA Technologies) was run using a QuantStudio 7 Flex Real-Time PCR System (Applied Biosystems). The PrimeTime assays used were as follows: *ARL13B*- Hs.PT.58.39963208, *ARL3*- Hs.PT.56a.40668589 and *UNC11B*- Hs.PT.58.38351751. Expression levels were normalised to the expression of housekeeping genes *HPRT1* and *GUSB*.

## Supplementary Material

Supplementary information

Reviewer comments

## References

[JCS258568C1] Alkanderi, S., Molinari, E., Shaheen, R., Elmaghloob, Y., Stephen, L. A., Sammut, V., Ramsbottom, S. A., Srivastava, S., Cairns, G., Edwards, N. et al. (2018). ARL3 mutations cause joubert syndrome by disrupting ciliary protein composition. *Am. J. Hum. Genet.* 103, 612-620. 10.1016/j.ajhg.2018.08.015 30269812 PMC6174286

[JCS258568C2] Anders, S., Pyl, P. T. and Huber, W. (2015). HTSeq--a Python framework to work with high-throughput sequencing data. *Bioinformatics* 31, 166-169. 10.1093/bioinformatics/btu638 25260700 PMC4287950

[JCS258568C3] Anderson, J. L., Mulligan, T. S., Shen, M.-C., Wang, H., Scahill, C. M., Tan, F. J., Du, S. J., Busch-Nentwich, E. M. and Farber, S. A. (2017). mRNA processing in mutant zebrafish lines generated by chemical and CRISPR-mediated mutagenesis produces unexpected transcripts that escape nonsense-mediated decay. *PLoS Genet.* 13, e1007105. 10.1371/journal.pgen.1007105 29161261 PMC5716581

[JCS258568C4] Baala, L., Audollent, S., Martinovic, J., Ozilou, C., Babron, M.-C., Sivanandamoorthy, S., Saunier, S., Salomon, R., Gonzales, M., Rattenberry, E. et al. (2007). Pleiotropic effects of CEP290 (NPHP6) mutations extend to Meckel syndrome. *Am. J. Hum. Genet.* 81, 170-179. 10.1086/519494 17564974 PMC1950929

[JCS258568C5] Bachmann-Gagescu, R. and Neuhauss, S. C. F. (2019). The photoreceptor cilium and its diseases. *Curr. Opin. Genet. Dev.* 56, 22-33. 10.1016/j.gde.2019.05.004 31260874

[JCS258568C6] Badano, J. L., Mitsuma, N., Beales, P. L. and Katsanis, N. (2006). The ciliopathies: an emerging class of human genetic disorders. *Annu. Rev. Genomics Hum. Genet.* 7, 125-148. 10.1146/annurev.genom.7.080505.115610 16722803

[JCS258568C7] Barny, I., Perrault, I., Michel, C., Soussan, M., Goudin, N., Rio, M., Thomas, S., Attié-Bitach, T., Hamel, C., Dollfus, H. et al. (2018). Basal exon skipping and nonsense-associated altered splicing allows bypassing complete CEP290 loss-of-function in individuals with unusually mild retinal disease. *Hum. Mol. Genet.* 27, 2689-2702. 10.1093/hmg/ddy179 29771326

[JCS258568C8] Barny, I., Perrault, I., Rio, M., Dollfus, H., Defoort-Dhellemmes, S., Kaplan, J., Rozet, J.-M. and Gerard, X. (2019). Description of two siblings with apparently severe CEP290 mutations and unusually mild retinal disease unrelated to basal exon skipping or nonsense-associated altered splicing. *Adv. Exp. Med. Biol.* 1185, 189-195. 10.1007/978-3-030-27378-1_31 31884610

[JCS258568C9] Baye, L. M., Patrinostro, X., Swaminathan, S., Beck, J. S., Zhang, Y., Stone, E. M., Sheffield, V. C. and Slusarski, D. C. (2011). The N-terminal region of centrosomal protein 290 (CEP290) restores vision in a zebrafish model of human blindness. *Hum. Mol. Genet.* 20, 1467-1477. 10.1093/hmg/ddr025 21257638 PMC3063982

[JCS258568C10] Bergboer, J. G. M., Wyatt, C., Austin-Tse, C., Yaksi, E. and Drummond, I. A. (2018). Assaying sensory ciliopathies using calcium biosensor expression in zebrafish ciliated olfactory neurons. *Cilia* 7, 2. 10.1186/s13630-018-0056-1 29568513 PMC5856005

[JCS258568C11] Bergen, D. J. M., Stevenson, N. L., Skinner, R. E. H., Stephens, D. J. and Hammond, C. L. (2017). The Golgi matrix protein giantin is required for normal cilia function in zebrafish. *Biol Open* 6, 1180-1189. 10.1242/bio.025502 28546340 PMC5576078

[JCS258568C12] Broekhuis, J. R., Leong, W. Y. and Jansen, G. (2013). Regulation of cilium length and intraflagellar transport. *Int. Rev. Cell Mol. Biol.* 303, 101-138. 10.1016/B978-0-12-407697-6.00003-9 23445809

[JCS258568C13] Buglo, E., Sarmiento, E., Martuscelli, N. B., Sant, D. W., Danzi, M. C., Abrams, A. J., Dallman, J. E. and Züchner, S. (2020). Genetic compensation in a stable slc25a46 mutant zebrafish: a case for using F0 CRISPR mutagenesis to study phenotypes caused by inherited disease. *PLoS ONE* 15, e0230566. 10.1371/journal.pone.0230566 32208444 PMC7092968

[JCS258568C14] Chang, B., Khanna, H., Hawes, N., Jimeno, D., He, S., Lillo, C., Parapuram, S. K., Cheng, H., Scott, A., Hurd, R. E. et al. (2006). In-frame deletion in a novel centrosomal/ciliary protein CEP290/NPHP6 perturbs its interaction with RPGR and results in early-onset retinal degeneration in the rd16 mouse. *Hum. Mol. Genet.* 15, 1847-1857. 10.1093/hmg/ddl107 16632484 PMC1592550

[JCS258568C15] Coppieters, F., Lefever, S., Leroy, B. P. and De Baere, E. (2010). CEP290, a gene with many faces: mutation overview and presentation of CEP290base. *Hum. Mutat.* 31, 1097-1108. 10.1002/humu.21337 20690115

[JCS258568C16] Craige, B., Tsao, C.-C., Diener, D. R., Hou, Y., Lechtreck, K.-F., Rosenbaum, J. L. and Witman, G. B. (2010). CEP290 tethers flagellar transition zone microtubules to the membrane and regulates flagellar protein content. *J. Cell Biol.* 190, 927-940. 10.1083/jcb.201006105 20819941 PMC2935561

[JCS258568C17] den Hollander, A. I., Koenekoop, R. K., Yzer, S., Lopez, I., Arends, M. L., Voesenek, K. E. J., Zonneveld, M. N., Strom, T. M., Meitinger, T., Brunner, H. G. et al. (2006). Mutations in the CEP290 (NPHP6) gene are a frequent cause of Leber congenital amaurosis. *Am. J. Hum. Genet.* 79, 556-561. 10.1086/507318 16909394 PMC1559533

[JCS258568C18] Dobin, A., Davis, C. A., Schlesinger, F., Drenkow, J., Zaleski, C., Jha, S., Batut, P., Chaisson, M. and Gingeras, T. R. (2013). STAR: ultrafast universal RNA-seq aligner. *Bioinformatics* 29, 15-21. 10.1093/bioinformatics/bts635 23104886 PMC3530905

[JCS258568C19] Drivas, T. G., Holzbaur, E. L. F. and Bennett, J. (2013). Disruption of CEP290 microtubule/membrane-binding domains causes retinal degeneration. *J. Clin. Invest.* 123, 4525-4539. 10.1172/JCI69448 24051377 PMC3784542

[JCS258568C20] Drivas, T. G., Wojno, A. P., Tucker, B. A., Stone, E. M. and Bennett, J. (2015). Basal exon skipping and genetic pleiotropy: a predictive model of disease pathogenesis. *Sci. Transl. Med.* 7, 291ra97. 10.1126/scitranslmed.aaa5370 PMC4486480 26062849

[JCS258568C92] Drummond, I. A., Majumdar, A., Hentschel, H., Elger, M., Solnica-Krezel, L., Schier, A. F., Neuhauss, S. C., Stemple, D. L., Zwartkruis, F., Rangini, Z. et al. (1998). Early development of the zebrafish pronephros and analysis of mutations affecting pronephric function. *Development* 125, 4655-4667. 10.1242/dev.125.23.4655 9806915

[JCS258568C21] Duldulao, N. A., Lee, S. and Sun, Z. (2009). Cilia localization is essential for in vivo functions of the Joubert syndrome protein Arl13b/Scorpion. *Development* 136, 4033-4042. 10.1242/dev.036350 19906870 PMC2778746

[JCS258568C22] El-Brolosy, M. A., Kontarakis, Z., Rossi, A., Kuenne, C., Günther, S., Fukuda, N., Kikhi, K., Boezio, G. L. M., Takacs, C. M., Lai, S.-L. et al. (2019). Genetic compensation triggered by mutant mRNA degradation. *Nature* 568, 193-197. 10.1038/s41586-019-1064-z 30944477 PMC6707827

[JCS258568C23] Fansa, E. K., Kösling, S. K., Zent, E., Wittinghofer, A. and Ismail, S. (2016). PDE6delta-mediated sorting of INPP5E into the cilium is determined by cargo-carrier affinity. *Nat. Commun.* 7, 11366. 10.1038/ncomms11366 27063844 PMC5512577

[JCS258568C24] Fisher, S., Kuna, D., Caspary, T., Kahn, R. A. and Sztul, E. (2020). ARF family GTPases with links to cilia. *Am. J. Physiol. Cell Physiol.* 319, C404-C418. 10.1152/ajpcell.00188.2020 32520609 PMC7500214

[JCS258568C25] Frank, V., den Hollander, A. I., Brüchle, N. O., Zonneveld, M. N., Nürnberg, G., Becker, C., Du Bois, G., Kendziorra, H., Roosing, S., Senderek, J. et al. (2008). Mutations of the CEP290 gene encoding a centrosomal protein cause Meckel-Gruber syndrome. *Hum. Mutat.* 29, 45-52. 10.1002/humu.20614 17705300

[JCS258568C26] Gorden, N. T., Arts, H. H., Parisi, M. A., Coene, K. L. M., Letteboer, S. J., F. van Beersum, S. E. C., Mans, D. A., Hikida, A., Eckert, M., Knutzen, D. et al. (2008). CC2D2A is mutated in Joubert syndrome and interacts with the ciliopathy-associated basal body protein CEP290. *Am. J. Hum. Genet.* 83, 559-571. 10.1016/j.ajhg.2008.10.002 18950740 PMC2668034

[JCS258568C27] Gotthardt, K., Lokaj, M., Koerner, C., Falk, N., Giessl, A. and Wittinghofer, A. (2015). A G-protein activation cascade from Arl13B to Arl3 and implications for ciliary targeting of lipidated proteins. *eLife* 4, e11859. 10.7554/eLife.11859 26551564 PMC4868535

[JCS258568C28] Hanke-Gogokhia, C., Wu, Z., Gerstner, C. D., Frederick, J. M., Zhang, H. and Baehr, W. (2016). Arf-like Protein 3 (ARL3) regulates protein trafficking and ciliogenesis in mouse photoreceptors. *J. Biol. Chem.* 291, 7142-7155. 10.1074/jbc.M115.710954 26814127 PMC4807295

[JCS258568C29] Huang, D. W., Sherman, B. T. and Lempicki, R. A. (2009). Systematic and integrative analysis of large gene lists using DAVID bioinformatics resources. *Nat. Protoc.* 4, 44-57. 10.1038/nprot.2008.211 19131956

[JCS258568C94] Humphrey, C. D. and Pittman, F. E. (1974). A simple methylene blue-azure II-basic fuchsin stain for epoxy-embedded tissue sections. *Stain Technol.* 49, 9-14. 10.3109/10520297409116929 4131626

[JCS258568C90] Hwang, W. Y., Fu, Y., Reyon, D., Maeder, M. L., Tsai, S. Q., Sander, J. D., Peterson, R. T., Yeh, J. R. and Joung, J. K. (2013). Efficient genome editing in zebrafish using a CRISPR-Cas system. *Nat. Biotechnol*. 31, 227-229. 10.1038/nbt.2501 23360964 PMC3686313

[JCS258568C30] Hynes, A. M., Giles, R. H., Srivastava, S., Eley, L., Whitehead, J., Danilenko, M., Raman, S., Slaats, G. G., Colville, J. G., Ajzenberg, H. et al. (2014). Murine Joubert syndrome reveals Hedgehog signaling defects as a potential therapeutic target for nephronophthisis. *Proc. Natl. Acad. Sci. USA* 111, 9893-9898. 10.1073/pnas.1322373111 24946806 PMC4103340

[JCS258568C31] Ishikawa, H., Thompson, J., Yates, J. R., III and Marshall, W. F. (2012). Proteomic analysis of mammalian primary cilia. *Curr. Biol.* 22, 414-419. 10.1016/j.cub.2012.01.031 22326026 PMC3298568

[JCS258568C32] Ismail, S. A., Chen, Y.-X., Rusinova, A., Chandra, A., Bierbaum, M., Gremer, L., Triola, G., Waldmann, H., Bastiaens, P. I. H. and Wittinghofer, A. (2011). Arl2-GTP and Arl3-GTP regulate a GDI-like transport system for farnesylated cargo. *Nat. Chem. Biol.* 7, 942-949. 10.1038/nchembio.686 22002721

[JCS258568C33] Ismail, S. A., Chen, Y.-X., Miertzschke, M., Vetter, I. R., Koerner, C. and Wittinghofer, A. (2012). Structural basis for Arl3-specific release of myristoylated ciliary cargo from UNC119. *EMBO J.* 31, 4085-4094. 10.1038/emboj.2012.257 22960633 PMC3474929

[JCS258568C34] Jaiswal, M., Fansa, E. K., Kösling, S. K., Mejuch, T., Waldmann, H. and Wittinghofer, A. (2016). Novel biochemical and structural insights into the interaction of myristoylated cargo with Unc119 protein and their release by Arl2/3. *J. Biol. Chem.* 291, 20766-20778. 10.1074/jbc.M116.741827 27481943 PMC5034065

[JCS258568C35] Jensen, V. L. and Leroux, M. R. (2017). Gates for soluble and membrane proteins, and two trafficking systems (IFT and LIFT), establish a dynamic ciliary signaling compartment. *Curr. Opin. Cell Biol.* 47, 83-91. 10.1016/j.ceb.2017.03.012 28432921

[JCS258568C36] Kim, J., Krishnaswami, S. R. and Gleeson, J. G. (2008). CEP290 interacts with the centriolar satellite component PCM-1 and is required for Rab8 localization to the primary cilium. *Hum. Mol. Genet.* 17, 3796-3805. 10.1093/hmg/ddn277 18772192 PMC2722899

[JCS258568C37] Lee, L. and Ostrowski, L. E. (2021). Motile cilia genetics and cell biology: big results from little mice. *Cell. Mol. Life Sci.* 78, 769-797. 10.1007/s00018-020-03633-5 32915243 PMC7902362

[JCS258568C38] Lessieur, E. M., Song, P., Nivar, G. C., Piccillo, E. M., Fogerty, J., Rozic, R. and Perkins, B. D. (2019). Ciliary genes arl13b, ahi1 and cc2d2a differentially modify expression of visual acuity phenotypes but do not enhance retinal degeneration due to mutation of cep290 in zebrafish. *PLoS ONE* 14, e0213960. 10.1371/journal.pone.0213960 30970040 PMC6457629

[JCS258568C39] Li, J. B., Gerdes, J. M., Haycraft, C. J., Fan, Y., Teslovich, T. M., May-Simera, H., Li, H., Blacque, O. E., Li, L., Leitch, C. C. et al. (2004). Comparative genomics identifies a flagellar and basal body proteome that includes the BBS5 human disease gene. *Cell* 117, 541-552. 10.1016/S0092-8674(04)00450-7 15137946

[JCS258568C40] Li, Y., Ling, K. and Hu, J. (2012). The emerging role of Arf/Arl small GTPases in cilia and ciliopathies. *J. Cell. Biochem.* 113, 2201-2207. 10.1002/jcb.24116 22389062 PMC4133128

[JCS258568C41] Littink, K. W., Pott, J.-W. R., Collin, R. W. J., Kroes, H. Y., Verheij, J. B. G. M., Blokland, E. A. W., de Castro Miró, M., Hoyng, C. B., Klaver, C. C. W., Koenekoop, R. K. et al. (2010). A novel nonsense mutation in CEP290 induces exon skipping and leads to a relatively mild retinal phenotype. *Invest. Ophthalmol. Vis. Sci.* 51, 3646-3652. 10.1167/iovs.09-5074 20130272

[JCS258568C42] Lopes, C. A. M., Prosser, S. L., Romio, L., Hirst, R. A., O'Callaghan, C., Woolf, A. S. and Fry, A. M. (2011). Centriolar satellites are assembly points for proteins implicated in human ciliopathies, including oral-facial-digital syndrome 1. *J. Cell Sci.* 124, 600-612. 10.1242/jcs.077156 21266464 PMC3031371

[JCS258568C43] Lu, H., Toh, M. T., Narasimhan, V., Thamilselvam, S. K., Choksi, S. P. and Roy, S. (2015). A function for the Joubert syndrome protein Arl13b in ciliary membrane extension and ciliary length regulation. *Dev. Biol.* 397, 225-236. 10.1016/j.ydbio.2014.11.009 25448689

[JCS258568C44] Ma, Z., Zhu, P., Shi, H., Guo, L., Zhang, Q., Chen, Y., Chen, S., Zhang, Z., Peng, J. and Chen, J. (2019). PTC-bearing mRNA elicits a genetic compensation response via Upf3a and COMPASS components. *Nature* 568, 259-263. 10.1038/s41586-019-1057-y 30944473

[JCS258568C45] Mayer, U., Küller, A., Daiber, P. C., Neudorf, I., Warnken, U., Schnölzer, M., Frings, S. and Möhrlen, F. (2009). The proteome of rat olfactory sensory cilia. *Proteomics* 9, 322-334. 10.1002/pmic.200800149 19086097

[JCS258568C46] McEwen, D. P., Koenekoop, R. K., Khanna, H., Jenkins, P. M., Lopez, I., Swaroop, A. and Martens, J. R. (2007). Hypomorphic CEP290/NPHP6 mutations result in anosmia caused by the selective loss of G proteins in cilia of olfactory sensory neurons. *Proc. Natl. Acad. Sci. USA* 104, 15917-15922. 10.1073/pnas.0704140104 17898177 PMC2000398

[JCS258568C47] Molinari, E., Srivastava, S., Dewhurst, R. M. and Sayer, J. A. (2020). Use of patient derived urine renal epithelial cells to confirm pathogenicity of PKHD1 alleles. *BMC Nephrol.* 21, 435. 10.1186/s12882-020-02094-z 33059616 PMC7559414

[JCS258568C48] Murga-Zamalloa, C. A., Ghosh, A. K., Patil, S. B., Reed, N. A., Chan, L. S., Davuluri, S., Peränen, J., Hurd, T. W., Rachel, R. A. and Khanna, H. (2011). Accumulation of the Raf-1 kinase inhibitory protein (Rkip) is associated with Cep290-mediated photoreceptor degeneration in ciliopathies. *J. Biol. Chem.* 286, 28276-28286. 10.1074/jbc.M111.237560 21685394 PMC3151072

[JCS258568C49] Nadeau, J. H. (2001). Modifier genes in mice and humans. *Nat. Rev. Genet.* 2, 165-174. 10.1038/35056009 11256068

[JCS258568C93] Nixon, S. J., Webb, R. I., Floetenmeyer, M., Schieber, N., Lo, H. P. and Parton, R. G. (2009). A single method for cryofixation and correlative light, electron microscopy and tomography of zebrafish embryos. *Traffic* 10, 131-136. 10.1111/j.1600-0854.2008.00859.x 19054388

[JCS258568C50] Nogales-Cadenas, R., Abascal, F., Diez-Perez, J., Carazo, J. M. and Pascual-Montano, A. (2009). CentrosomeDB: a human centrosomal proteins database. *Nucleic Acids Res.* 37, D175-D180. 10.1093/nar/gkn815 18971254 PMC2686521

[JCS258568C51] Nozaki, S., Katoh, Y., Terada, M., Michisaka, S., Funabashi, T., Takahashi, S., Kontani, K. and Nakayama, K. (2017). Regulation of ciliary retrograde protein trafficking by the Joubert syndrome proteins ARL13B and INPP5E. *J. Cell Sci.* 130, 563-576. 10.1242/jcs.197004 27927754

[JCS258568C52] Panizzi, J. R., Becker-Heck, A., Castleman, V. H., Al-Mutairi, D. A., Liu, Y., Loges, N. T., Pathak, N., Austin-Tse, C., Sheridan, E., Schmidts, M. et al. (2012). CCDC103 mutations cause primary ciliary dyskinesia by disrupting assembly of ciliary dynein arms. *Nat. Genet.* 44, 714-719. 10.1038/ng.2277 22581229 PMC3371652

[JCS258568C53] Pazour, G. J., Agrin, N., Leszyk, J. and Witman, G. B. (2005). Proteomic analysis of a eukaryotic cilium. *J. Cell Biol.* 170, 103-113. 10.1083/jcb.200504008 15998802 PMC2171396

[JCS258568C54] Powell, L., Samarakoon, Y. H., Ismail, S. and Sayer, J. A. (2019). ARL3, a small GTPase with a functionally conserved role in primary cilia and immune synapses. *Small GTPases* 12, 167-176. 10.1080/21541248.2019.1703466 31826708 PMC7939558

[JCS258568C55] Prevo, B., Scholey, J. M. and Peterman, E. J. G. (2017). Intraflagellar transport: mechanisms of motor action, cooperation, and cargo delivery. *FEBS J.* 284, 2905-2931. 10.1111/febs.14068 28342295 PMC5603355

[JCS258568C56] Rachel, R. A., May-Simera, H. L., Veleri, S., Gotoh, N., Choi, B. Y., Murga-Zamalloa, C., McIntyre, J. C., Marek, J., Lopez, I., Hackett, A. N. et al. (2012). Combining Cep290 and Mkks ciliopathy alleles in mice rescues sensory defects and restores ciliogenesis. *J. Clin. Invest.* 122, 1233-1245. 10.1172/JCI60981 22446187 PMC3314468

[JCS258568C57] Rachel, R. A., Yamamoto, E. A., Dewanjee, M. K., May-Simera, H. L., Sergeev, Y. V., Hackett, A. N., Pohida, K., Munasinghe, J., Gotoh, N., Wickstead, B. et al. (2015). CEP290 alleles in mice disrupt tissue-specific cilia biogenesis and recapitulate features of syndromic ciliopathies. *Hum. Mol. Genet.* 24, 3775-3791. 10.1093/hmg/ddv123 25859007 PMC4459394

[JCS258568C58] Ramsbottom, S. A., Thelwall, P. E., Wood, K. M., Clowry, G. J., Devlin, L. A., Silbermann, F., Spiewak, H. L., Shril, S., Molinari, E., Hildebrandt, F. et al. (2020). Mouse genetics reveals Barttin as a genetic modifier of Joubert syndrome. *Proc. Natl. Acad. Sci. USA* 117, 1113-1118. 10.1073/pnas.1912602117 31879347 PMC6969532

[JCS258568C59] Rao, K. N., Zhang, W., Li, L., Ronquillo, C., Baehr, W. and Khanna, H. (2016). Ciliopathy-associated protein CEP290 modifies the severity of retinal degeneration due to loss of RPGR. *Hum. Mol. Genet.* 25, 2005-2012. 10.1093/hmg/ddw075 26936822 PMC5062589

[JCS258568C60] Reiter, J. F. and Leroux, M. R. (2017). Genes and molecular pathways underpinning ciliopathies. *Nat. Rev. Mol. Cell Biol.* 18, 533-547. 10.1038/nrm.2017.60 28698599 PMC5851292

[JCS258568C61] Revenkova, E., Liu, Q., Gusella, G. L. and Iomini, C. (2018). The Joubert syndrome protein ARL13B binds tubulin to maintain uniform distribution of proteins along the ciliary membrane. *J. Cell Sci.* 131, jcs212324. 10.1242/jcs.212324 29592971 PMC5992585

[JCS258568C62] Robinson, M. D., McCarthy, D. J. and Smyth, G. K. (2010). edgeR: a Bioconductor package for differential expression analysis of digital gene expression data. *Bioinformatics* 26, 139-140. 10.1093/bioinformatics/btp616 19910308 PMC2796818

[JCS258568C63] Rossi, A., Kontarakis, Z., Gerri, C., Nolte, H., Hölper, S., Krüger, M. and Stainier, D. Y. R. (2015). Genetic compensation induced by deleterious mutations but not gene knockdowns. *Nature* 524, 230-233. 10.1038/nature14580 26168398

[JCS258568C64] Roy, K. and Marin, E. P. (2019). Lipid modifications in cilia biology. *J. Clin. Med.* 8, 921. 10.3390/jcm8070921 PMC6678300 31252577

[JCS258568C65] Sang, L., Miller, J. J., Corbit, K. C., Giles, R. H., Brauer, M. J., Otto, E. A., Baye, L. M., Wen, X., Scales, S. J., Kwong, M. et al. (2011). Mapping the NPHP-JBTS-MKS protein network reveals ciliopathy disease genes and pathways. *Cell* 145, 513-528. 10.1016/j.cell.2011.04.019 21565611 PMC3383065

[JCS258568C66] Sayer, J. A., Otto, E. A., O'Toole, J. F., Nurnberg, G., Kennedy, M. A., Becker, C., Hennies, H. C., Helou, J., Attanasio, M., Fausett, B. V. et al. (2006). The centrosomal protein nephrocystin-6 is mutated in Joubert syndrome and activates transcription factor ATF4. *Nat. Genet.* 38, 674-681. 10.1038/ng1786 16682973

[JCS258568C67] Schäfer, T., Pütz, M., Lienkamp, S., Ganner, A., Bergbreiter, A., Ramachandran, H., Gieloff, V., Gerner, M., Mattonet, C., Czarnecki, P. G. et al. (2008). Genetic and physical interaction between the NPHP5 and NPHP6 gene products. *Hum. Mol. Genet.* 17, 3655-3662. 10.1093/hmg/ddn260 18723859 PMC2802281

[JCS258568C68] Schrick, J. J., Vogel, P., Abuin, A., Hampton, B. and Rice, D. S. (2006). ADP-ribosylation factor-like 3 is involved in kidney and photoreceptor development. *Am. J. Pathol.* 168, 1288-1298. 10.2353/ajpath.2006.050941 16565502 PMC1606550

[JCS258568C69] Serobyan, V., Kontarakis, Z., El-Brolosy, M. A., Welker, J. M., Tolstenkov, O., Saadeldein, A. M., Retzer, N., Gottschalk, A., Wehman, A. M. and Stainier, D. Y. R. (2020). Transcriptional adaptation in Caenorhabditis elegans. *eLife* 9, e50014. 10.7554/eLife.50014 31951195 PMC6968918

[JCS258568C70] Shimada, H., Lu, Q., Insinna-Kettenhofen, C., Nagashima, K., English, M. A., Semler, E. M., Mahgerefteh, J., Cideciyan, A. V., Li, T., Brooks, B. P. et al. (2017). In vitro modeling using ciliopathy-patient-derived cells reveals distinct cilia dysfunctions caused by CEP290 mutations. *Cell Rep* 20, 384-396. 10.1016/j.celrep.2017.06.045 28700940 PMC5553702

[JCS258568C71] Song, P., Dudinsky, L., Fogerty, J., Gaivin, R. and Perkins, B. D. (2016). Arl13b Interacts With Vangl2 to Regulate Cilia and Photoreceptor Outer Segment Length in Zebrafish. *Invest. Ophthalmol. Vis. Sci.* 57, 4517-4526. 10.1167/iovs.16-19898 27571019 PMC5015978

[JCS258568C72] Stawicki, T. M., Hernandez, L., Esterberg, R., Linbo, T., Owens, K. N., Shah, A. N., Thapa, N., Roberts, B., Moens, C. B., Rubel, E. W. et al. (2016). Cilia-associated genes play differing roles in aminoglycoside-induced hair cell death in Zebrafish. *G3 (Bethesda)* 6, 2225-2235. 10.1534/g3.116.030080 27207957 PMC4938675

[JCS258568C73] Stevenson, N. L., Bergen, D. J. M., Skinner, R. E. H., Kague, E., Martin-Silverstone, E., Robson Brown, K. A., Hammond, C. L. and Stephens, D. J. (2017). Giantin-knockout models reveal a feedback loop between Golgi function and glycosyltransferase expression. *J. Cell Sci.* 130, 4132-4143. 10.1242/jcs.212308 29093022 PMC5769581

[JCS258568C74] Stevenson, N. L., Bergen, D. J. M., Xu, A., Wyatt, E., Henry, F., McCaughey, J., Vuolo, L., Hammond, C. L. and Stephens, D. J. (2018). Regulator of calcineurin-2 is a centriolar protein with a role in cilia length control. *J. Cell Sci.* 131, jcs212258. 10.1242/jcs.212258 29643119 PMC5992583

[JCS258568C75] Stowe, T. R., Wilkinson, C. J., Iqbal, A. and Stearns, T. (2012). The centriolar satellite proteins Cep72 and Cep290 interact and are required for recruitment of BBS proteins to the cilium. *Mol. Biol. Cell* 23, 3322-3335. 10.1091/mbc.e12-02-0134 22767577 PMC3431927

[JCS258568C76] Sztal, T. E. and Stainier, D. Y. R. (2020). Transcriptional adaptation: a mechanism underlying genetic robustness. *Development* 147, dev186452. 10.1242/dev.186452 32816903

[JCS258568C77] Sztal, T. E., McKaige, E. A., Williams, C., Ruparelia, A. A. and Bryson-Richardson, R. J. (2018). Genetic compensation triggered by actin mutation prevents the muscle damage caused by loss of actin protein. *PLoS Genet.* 14, e1007212. 10.1371/journal.pgen.1007212 29420541 PMC5821405

[JCS258568C78] Tang, Q., Iyer, S., Lobbardi, R., Moore, J. C., Chen, H., Lareau, C., Hebert, C., Shaw, M. K. L., Neftel, C., Suva, M. L. et al. (2017). Dissecting hematopoietic and renal cell heterogeneity in adult zebrafish at single-cell resolution using RNA sequencing. *J. Exp. Med.* 214, 2875-2887. 10.1084/jem.20170976 28878000 PMC5626406

[JCS258568C79] Thomas, S., Wright, K. J., Le Corre, S., Micalizzi, A., Romani, M., Abhyankar, A., Saada, J., Perrault, I., Amiel, J., Litzler, J. et al. (2014). A homozygous PDE6D mutation in Joubert syndrome impairs targeting of farnesylated INPP5E protein to the primary cilium. *Hum. Mutat.* 35, 137-146. 10.1002/humu.22470 24166846 PMC3946372

[JCS258568C80] Tsang, W. Y., Bossard, C., Khanna, H., Peränen, J., Swaroop, A., Malhotra, V. and Dynlacht, B. D. (2008). CP110 suppresses primary cilia formation through its interaction with CEP290, a protein deficient in human ciliary disease. *Dev. Cell* 15, 187-197. 10.1016/j.devcel.2008.07.004 18694559 PMC3987787

[JCS258568C81] Tsujikawa, M. and Malicki, J. (2004). Intraflagellar transport genes are essential for differentiation and survival of vertebrate sensory neurons. *Neuron* 42, 703-716. 10.1016/S0896-6273(04)00268-5 15182712

[JCS258568C82] Valente, E. M., Silhavy, J. L., Brancati, F., Barrano, G., Krishnaswami, S. R., Castori, M., Lancaster, M. A., Boltshauser, E., Boccone, L., Al-Gazali, L. et al. (2006). Mutations in CEP290, which encodes a centrosomal protein, cause pleiotropic forms of Joubert syndrome. *Nat. Genet.* 38, 623-625. 10.1038/ng1805 16682970

[JCS258568C83] van Dam, T. J. P., Kennedy, J., van der Lee, R., de Vrieze, E., Wunderlich, K. A., Rix, S., Dougherty, G. W., Lambacher, N. J., Li, C., Jensen, V. L. et al. (2019). CiliaCarta: an integrated and validated compendium of ciliary genes. *PLoS ONE* 14, e0216705. 31095607 10.1371/journal.pone.0216705 PMC6522010

[JCS258568C84] Westerfield, M. (2000). *The Zebrafish Book. A Guide for the Laboratory use of Zebrafish (Danio rerio)*. Eugene: University of Oregon Press.

[JCS258568C91] Willems, E., Leyns, L. and Vandesompele, J. (2008). Standardization of real-time PCR gene expression data from independent biological replicates. *Anal Biochem.* 379, 127-129. 10.1016/j.ab.2008.04.036 18485881

[JCS258568C85] Williams, C. L., Li, C., Kida, K., Inglis, P. N., Mohan, S., Semenec, L., Bialas, N. J., Stupay, R. M., Chen, N., Blacque, O. E. et al. (2011). MKS and NPHP modules cooperate to establish basal body/transition zone membrane associations and ciliary gate function during ciliogenesis. *J. Cell Biol.* 192, 1023-1041. 10.1083/jcb.201012116 21422230 PMC3063147

[JCS258568C86] Wright, K. J., Baye, L. M., Olivier-Mason, A., Mukhopadhyay, S., Sang, L., Kwong, M., Wang, W., Pretorius, P. R., Sheffield, V. C., Sengupta, P. et al. (2011). An ARL3-UNC119-RP2 GTPase cycle targets myristoylated NPHP3 to the primary cilium. *Genes Dev.* 25, 2347-2360. 10.1101/gad.173443.111 22085962 PMC3222901

[JCS258568C87] Wu, Z., Pang, N., Zhang, Y., Chen, H., Peng, Y., Fu, J. and Wei, Q. (2020). CEP290 is essential for the initiation of ciliary transition zone assembly. *PLoS Biol.* 18, e3001034. 10.1371/journal.pbio.3001034 33370260 PMC7793253

[JCS258568C88] Zhang, Q., Li, Y., Zhang, Y., Torres, V. E., Harris, P. C., Ling, K. and Hu, J. (2016). GTP-binding of ARL-3 is activated by ARL-13 as a GEF and stabilized by UNC-119. *Sci. Rep.* 6, 24534. 10.1038/srep24534 27102355 PMC4840320

[JCS258568C89] Zhou, C., Cunningham, L., Marcus, A. I., Li, Y. and Kahn, R. A. (2006). Arl2 and Arl3 regulate different microtubule-dependent processes. *Mol. Biol. Cell* 17, 2476-2487. 10.1091/mbc.e05-10-0929 16525022 PMC1446103

