# Supplementary material for: Genetic compensation for cilia defects in cep290 mutants by upregulation of cilia-associated small GTPases
Source: J Cell Sci. 2021 Jul 22;134(14):jcs258568. doi: 10.1242/jcs.258568 (PMC8325957; doi:10.1242/jcs.258568)
Supplement: Supplementary information [file joces-134-258568-s1.pdf]

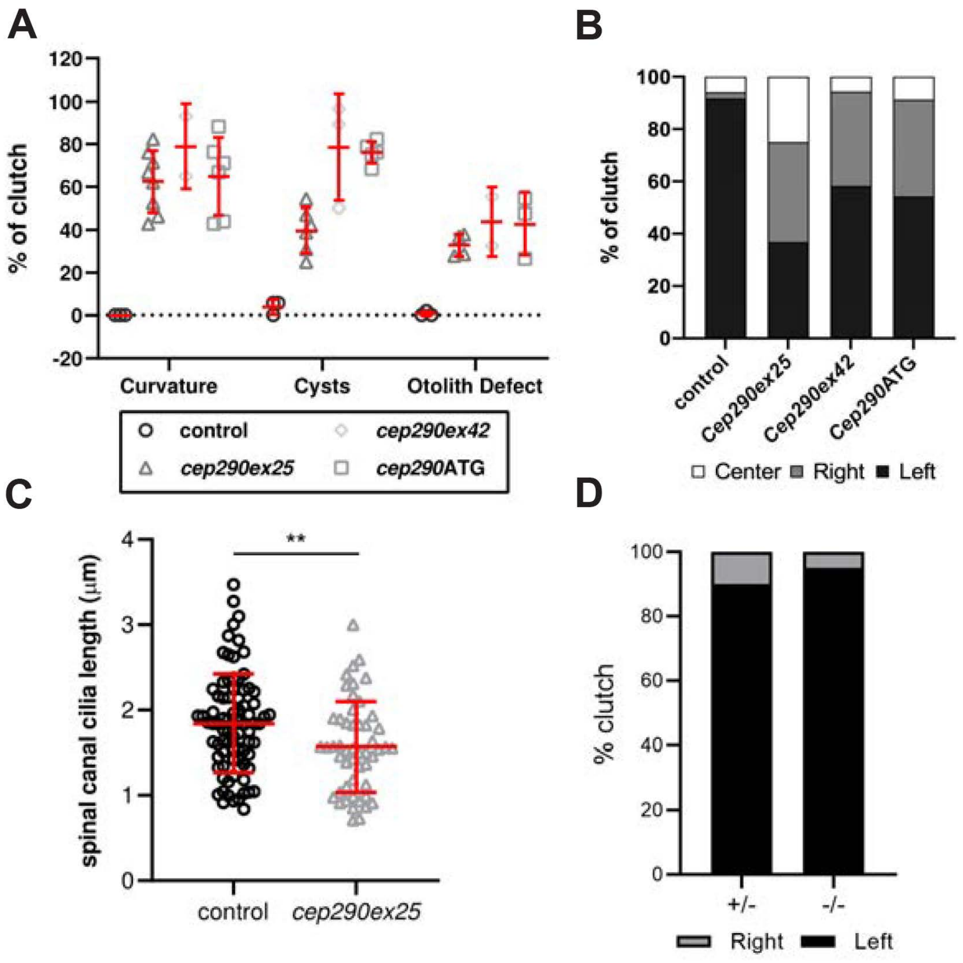

Figure S1. Cep290 knockdown cause cilia associated phenotypes.
A. Quantification of axis curvature, pronephric cysts, and otolith defects observed in 2dpf embryos injected with *cep290* and control morpholinos. Values are averages from at least two injected clutches (20-30 embryos/clutch). Error bars are SEM. **B.** Quantification of heart looping morphogenesis in 27hpf zebrafish embryos. Wildtype heart tubes looped towards the left, while embryos injected with *cep290* morpholinos exhibited randomized heart looping (to the left, right, or center). **C.** Quantification of spinal canal cilia length. *Cep290ex25* morphant embryos present shorter spinal canal cilia compared to stage-matched embryos injected with control morpholino. **D.** No significant situs inversus phenotype was observed in MZCep290 -/- embryos.

Journal of Cell Science • Supplementary information

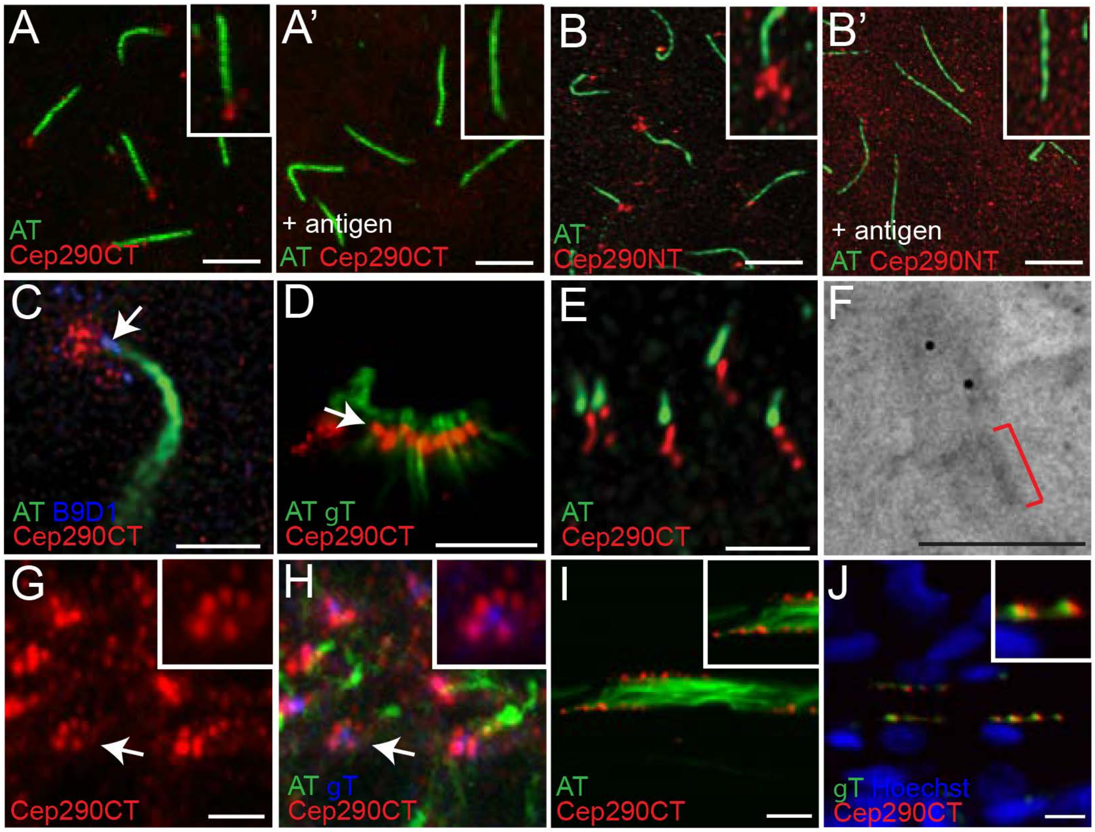

Journal of Cell Science • Supplementary information

Figure S2. Zebrafish Cep290 localizes to the transition zone and/or centriolar satellites in a tissue-specific manner.

In a 10 somite stage (ss) embryo, the Cep290CT (**A**) and NT (**B**) antibodies (red) stain punctate structures at the base of KV cilia (AT, green). Staining is lost when the antibodies are preincubated with antigen (A', B'), demonstrating antibody specificity. Insets: magnified view of a single cilia and basal body region. **C.** A magnified image of a single Kupffer's vesicle cilium (green) shows that Cep290CT (red) stain does not overlap (arrow) with the transition zone marker B9D1 (blue), indicating a pericentriolar localization. **D**. Cep290CT staining of 3dpf olfactory placode multiciliated cells. Cep290 localizes in a single lobe between acetylated tubulin (AT) and gamma-tubulin (γT) signals (both green). **E**. Cep290CT (red) staining of 3dpf photoreceptor connecting cilia. Cep290 is found in 2-3 distinct lobes, indicating transition zone and pericentriolar localization. **F**. Immuno-EM showing Cep290CT staining of photoreceptor connecting cilia. Post-embedding staining was performed on ultrathin sections cut from an embryo fixed by high pressure freezing. Red bracket indicates position of the basal body. **G-H.** Pericentriolar Cep290CT staining **(G)** is also observed at the base of spinal canal cilia **(H)**. Insets: magnified view of a single basal body region showing Cep290CT signal is found in close proximity to gamma tubulin. **I-J**. Cep290CT staining of pronephric tubules where Cep290 localizes in the base of pronephric cilia bundles **(I)** and adjacent but no overlapping with gamma-tubulin **(J)**, indicating transition zone localization. Anterior-posterior left-right orientation. Scale bars: **A,B,I,J** 5 µm; **C,D,E,G** 2 µm; **F** 0.5 µm.

Journal of Cell Science • Supplementary information

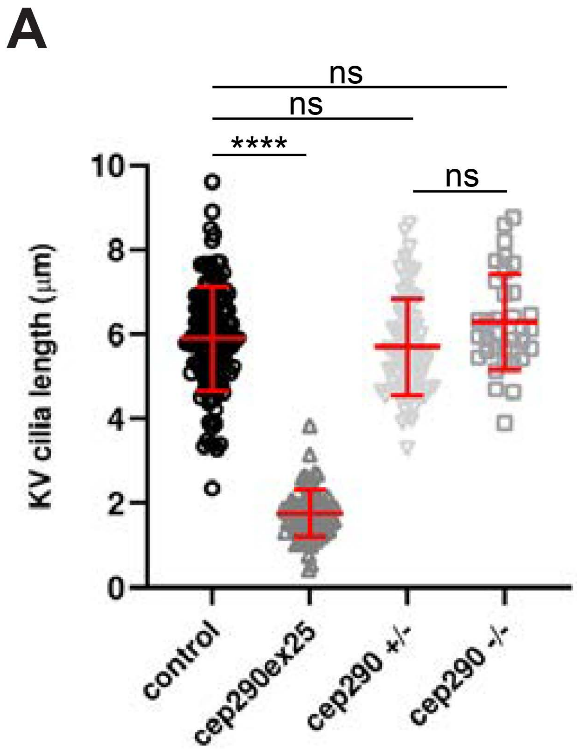

Figure S3. Acute vs long term loss of *cep290* differentially affects Kupffer's vesicle cilia. A. Quantification of KV cilia length. KV cilia are significantly shorter in *cep290ex25* morphant embryos as compared to embryos injected with control morpholino. Cep290 MZ mutant embryos did not show shorter KV cilia compared with heterozygous sibling embryos. n = >100 cilia from at least 3-5 embryos per condition. Error bars indicate SD. **** = p<0.0001 as determined by Tukey's test.

Journal of Cell Science • Supplementary information

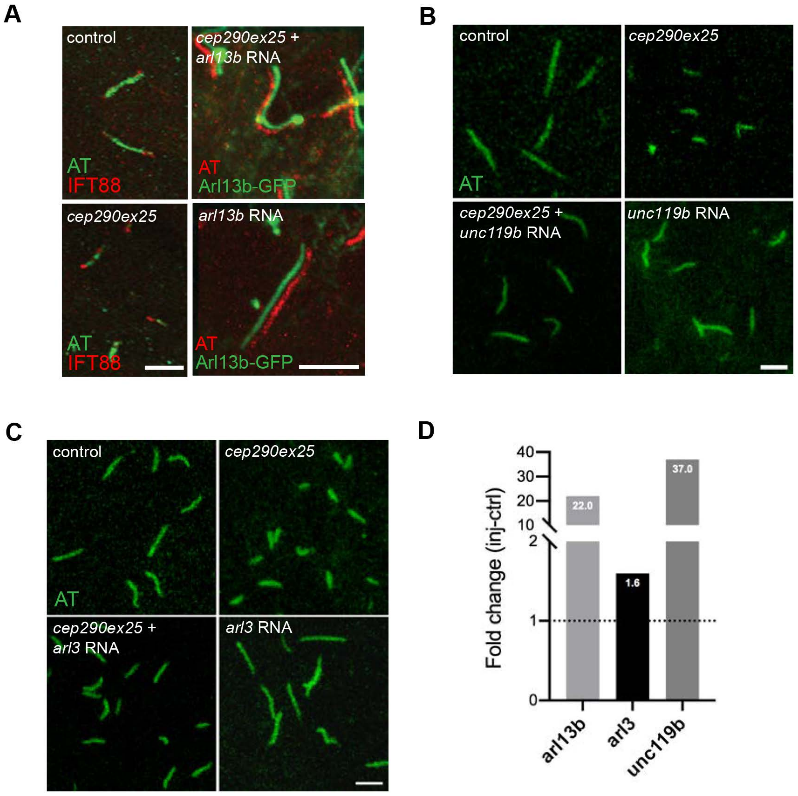

Figure S4. Upregulation of *arl3*, *arl13b* and *unc119b* rescue cep290ex25 Kupffer's vesicle cilia defects.

A-C. Representatives images of *cep290ex25* KV cilia rescued by injection of *arl13b* mRNA **(A)**, *unc119b* mRNA **(B)** or arl3 mRNA **(C)**. **D.** RT-qPCR from 48hpf WT embryos injected with *arl13b* mRNA, unc119b mRNA or *arl3* mRNA to confirm injection and stability of the mRNA. Scale bars: **A,B,C**, 5 µm.

Journal of Cell Science • Supplementary information

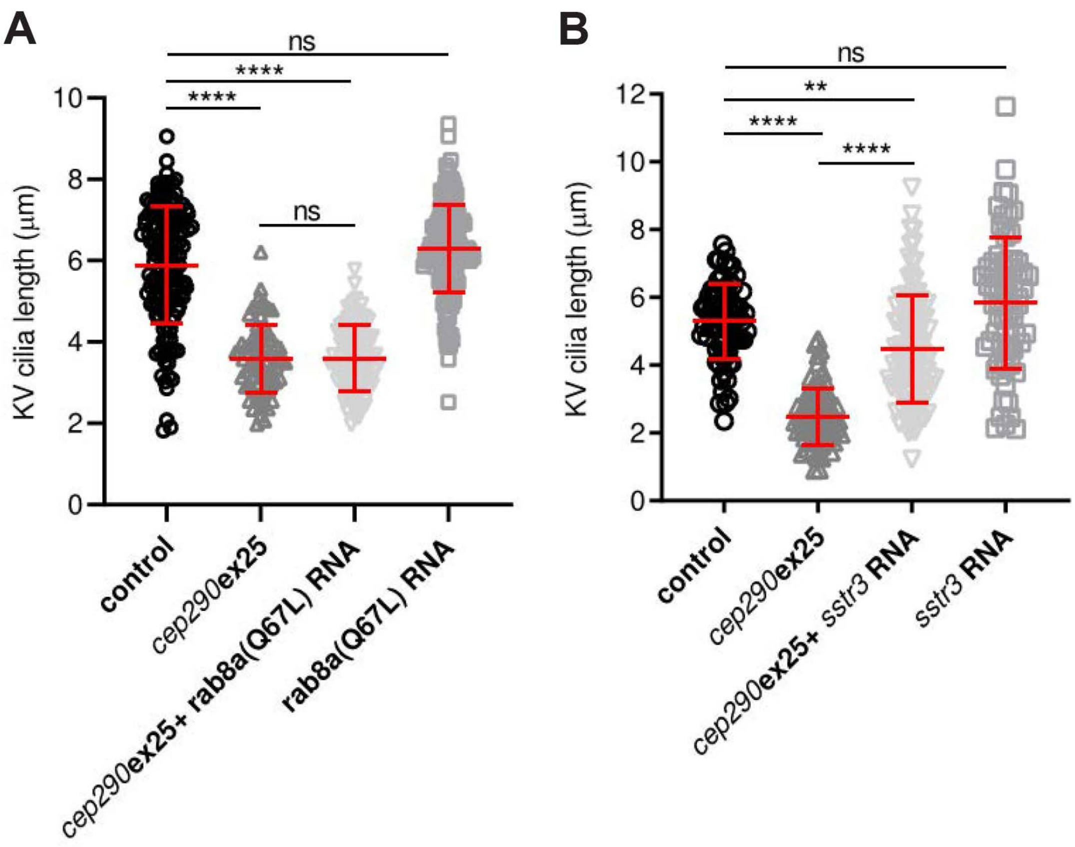

Figure S5. Expression of *rab8* does not rescue cep290ex25 Kupffer's vesicle cilia defects while *sstr3* expression does.

A. Injection of an activated rab8(Q67L) mRNA did not affect cilia length in *cep290ex25* morphant embryos. **B.** Injection of mRNA encoding the cilia-targeted GPCR *sstr3* rescued cilia length in *cep290ex25* morphant embryos suggesting that ectopically driving cilia membrane delivery can overcome deficiencies in cilia protein or membrane transport and affect cilia length.

Journal of Cell Science • Supplementary information

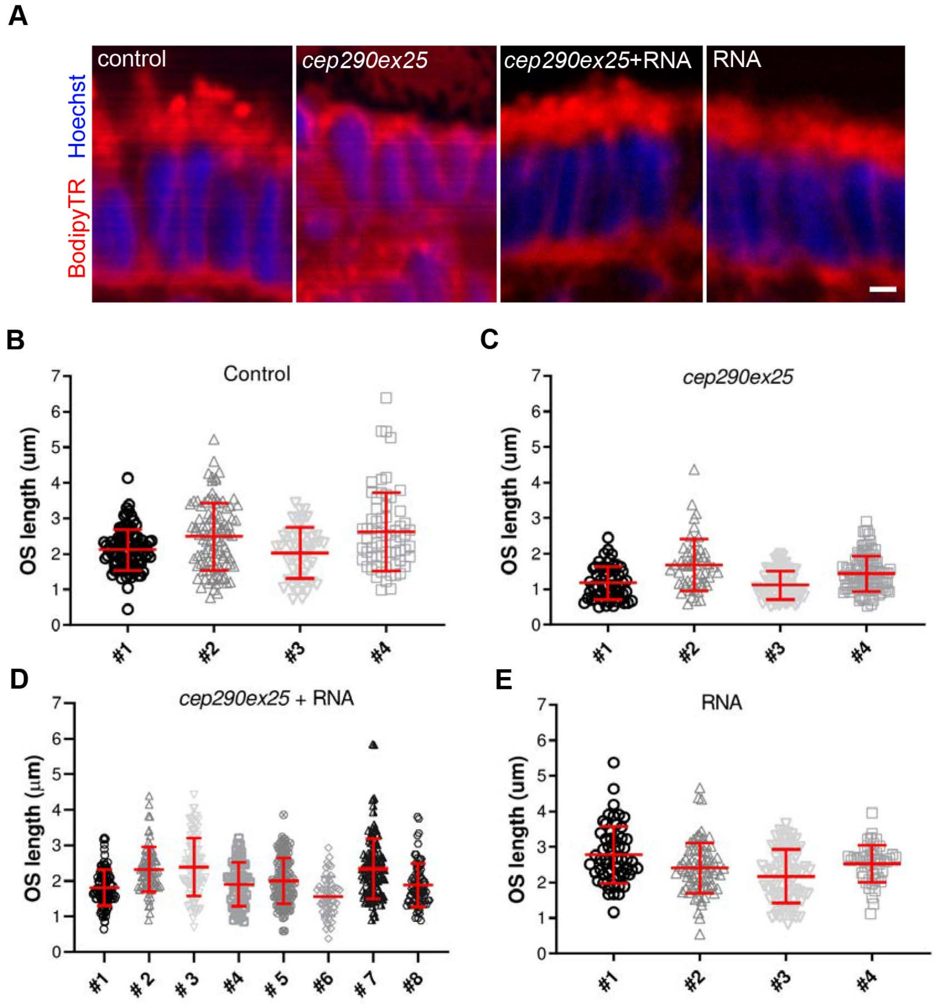

Figure S6. Photoreceptor outer segment length defect on *cep290ex25* morphants is rescued by upregulation of *arl3, arl13b* and *unc119b*.

A. Representative images of 3dpf photoreceptor outer segment length rescued by injection of *arl3*, *arl13b* and *unc119b* mRNA. **B-E**. Individual analysis of the OS length of control (**B**), *cep290ex25* (**C**), *cep290ex25* + RNA (**D**) and RNA (**E**) embryos showing that all the analyzed embryos in each group showed similar average of the OS length. Scale bar 2 µm.

Table S1. Differentially expressed genes between wildtype and *MZcep290*$^{fb208/fb208}$ mutant homozygotes at 3 dpf.

Click here to download Table S1

Table S2. Differentially expressed genes between *MZcep290*$^{fb208/fb208}$ mutant homozygotes and *cep290* morphants at 3 dpf.

Click here to download Table S2

Table S3. Differentially expressed genes between wildtype and *cep290* morphants at 3 dpf.

Click here to download Table S3

Table S4. Functional annotation of genes upregulated in *MZcep290*$^{fb208/fb208}$ mutant homozygotes vs. wildtype using the DAVID platform.

Click here to download Table S4

Table S5. Summary of cilia proteome candidate genes compensating for *cep290* deficiency.

Click here to download Table S5

Table S6. Oligonucleotide DNA primers used in analysis of mutants and morphants.

Click here to download Table S6

Journal of Cell Science • Supplementary information
