# Supplementary material for: Genetic compensation for cilia defects in cep290 mutants by upregulation of cilia-associated small GTPases
Source: J Cell Sci. 2021 Jul 22;134(14):jcs258568. doi: 10.1242/jcs.258568 (PMC8325957; doi:10.1242/jcs.258568)
Supplement: Reviewer comments [file joces-134-258568_review_history.pdf]

Journal of Cell Science

Genetic compensation for cilia defects in *cep290* mutants by upregulation of cilia-associated small GTPases

Magdalena Cardenas-Rodriguez, Christina Austin-Tse, Judith G.M. Bergboer, Elisa Molinari, Yuya Sugano, Ruxandra Bachmann-Gagescu, John A. Sayer and Iain A. Drummond
DOI: 10.1242/jcs.258568

Editor: David Stephens

Review timeline

Original submission:	24 February 2021
Editorial decision:	1 April 2021
First revision received:	26 May 2021
Accepted:	2 June 2021

Original submission

First decision letter

MS ID#: JOCES/2021/258568

MS TITLE: Genetic compensation for cilia defects in cep290/NPHP6 mutants by upregulation of cilia-associated small GTPases

AUTHORS: Magdalena Cardenas-Rodriguez, Christina Austin-Tse, Judith G.M Bergboer, Elisa Molinari, Yuya Sugano, Ruxandra Bachmann-Gagescu, John A Sayer, and Iain A Drummond
ARTICLE TYPE: Research Article

We have now reached a decision on the above manuscript.

To see the reviewers' reports and a copy of this decision letter, please go to: https://submit-jcs.biologists.org and click on the 'Manuscripts with Decisions' queue in the Author Area. (Corresponding author only has access to reviews.)

As you will see, the reviewers raise a number of criticisms that prevent me from accepting the paper at this stage. They suggest, however, that a revised version might prove acceptable, if you can address their concerns. If you think that you can deal satisfactorily with the criticisms on revision, I would be pleased to see a revised manuscript. We may then return it to the reviewers.

Overall the critique is relatively minor with only a few suggestions for further experimental work. For clarity, I consider that morpholino knockdown of unc119b and arl13b would not be necessary as these experiments are not likely to produce clearly defined outcomes. Thesuggestion totitrate CEP290 and control morpholinos to determine the timing of compensation is interesting and worth considering. However, I would not absolutely require this in revision as I am not convinced that it will substantially alter our level of understanding. The one issue that both reviewers (and indeed I) agree on is the need to share the full RNAseq data set. Indeed, this is a core policy of the journal (https://jcs.biologists.org/content/journal-policies#data). Specifically, "primary data for high-throughput experiments such as microarrays, RNA-seq, ChIP-chip or ChIP-seq be deposited in the appropriate public database. The Gene Expression Omnibus (GEO), ArrayExpress, European Nucleotide Archive (ENA) or Short Read Archive (SRA) are the appropriate repositories for most functional genomics data". Please provide the accession number for the raw data when deposited and include a summary file with the manuscript as suggested.

© 2021. Published by The Company of Biologists under the terms of the Creative Commons Attribution License (https://creativecommons.org/licenses/by/4.0/).

We are aware that you may be experiencing disruption to the normal running of your lab that makes experimental revisions challenging. If it would be helpful, we encourage you to contact us to discuss your revision in greater detail. Please send us a point-by-point response indicating where you are able to address concerns raised (either experimentally or by changes to the text) and where you will not be able to do so within the normal timeframe of a revision. We will then provide further guidance. Please also note that we are happy to extend revision timeframes as necessary.

Please ensure that you clearly highlight all changes made in the revised manuscript. Please avoid using 'Tracked changes' in Word files as these are lost in PDF conversion.

I should be grateful if you would also provide a point-by-point response detailing how you have dealt with the points raised by the reviewers in the 'Response to Reviewers' box. Please attend to all of the reviewers' comments. If you do not agree with any of their criticisms or suggestions please explain clearly why this is so.

Reviewer 1

Advance summary and potential significance to field

The authors describe zebrafish cep290 morphants and mutants and show that phenotypes are different which are likely due to compensation of small GTPases arl2, arl13 and unc119b. The authors provide strong data to support their claims and it could be of interest and significance to the field to further unravel compensatory mechanisms upon permanent removal of a genetic factor. It also sheds light on how cep290 may be chaperoned to fulfil it's ciliary function.

Comments for the author

This is a very nice paper and it was a good read. I do not have any major concerns about the design of the study, and this manuscript has included some interesting data that would be valuable to the cilia and zebrafish community. However, some things I was not able to verify such as statistical methods and catalogue numbers of antibodies used. I would also urge the authors to release the summary statistics RNA-seq data file as it was difficult for me to verify the statements regarding figure 5.

I would like to see some minor changes to some sections and I suggest a couple of experiments to further strengthen the manuscript.

Introduction:
Expand the on the human syndromes and associated phenotypes CEP290 LOF mutations causes.
A few lines are needed to explain the difference between primary, multi and motile cilia and whether cep290 is specifically localising to that.

Results In the results, please elaborate a bit more why exon 25 and exon 42 were selected for morpholino knockdown. Why was exon 16 selected for CRISPR/Cas9 mutagenesis?

In results, discussing figure 5B highlight (‘revealed a set 1,953 genes upregulated > 2-fold in MZcep290-/- mutants’) where the 1953 genes were 2 fold change was derived from. There are no supplemental data files (Excel file) with the baseline and differential expression data, so I am not in the position to check this claim. This will need to be supplied in the revisions. I encourage the authors to find a way to display the gene ontology analysis (i.e. GOrilla, STRING Network analysis, KEGG etc.) and publish it as a supplemental figure and make the data files associated with these analyses available.

It is strongly encouraged to perform a morpholino (cep290ex25 and control) titration experiment where e.g. half and a quarter of the original dosages are injected to determine ciliopathy frequency and subsequent protein expression analysis (i.e. 50% ex25 + 50%ctrlMO; 25%ex25 +75%ctrlMO mixtures). This to determine when morpholino and mutant would display a similar

© 2021. Published by The Company of Biologists under the terms of the Creative Commons Attribution License (https://creativecommons.org/licenses/by/4.0/).

phenotype and it would provide information on when the compensatory mechanism kicks in. This could be further bolstered with co-injecting RNAs the arl and unc mRNAs and/or qPCR expression analysis.

A morpholino knockdown of unc119b and arl13b in the mutants should induce a worse phenotype as it disrupts the compensatory mechanism (if there is a genetic interaction). This is a recommended experiment and if it is not feasible it should be discussed in the discussion as a future directions statement.

Discussion:
Add a couple of sentences to discuss / summarize the correlation and discrepancies of the zebrafish mutant phenotype and human/mouse phenotypes.

The authors discuss sclc25a46 and CBPA, there are also studies of golgb1 knockdown/mutant compensation via glycosyltransferases and RCAN2 (PMID: 29643119; PMID: 29093022; PMID: 28546340). These should be added.

Arl13b is known to regulate vangl2 and wnt signalling planar cell polarity pathway, could a disruption of this be repaired due to overexpression and hence the reduction of the ciliopathy phenotype in rescue experiments? This needs to be discussed (PMID: 27571019 already cited; PMID: 20305649)

Material methods:
Statistical methods should be provided in a separate heading including software used to calculate p-values RNA-seq analysis needs to be elaborated on which illumina platform and what bioinformatics pipeline was used to map the reads onto the zebrafish genome and how differential expression was calculated Catalogue numbers of commercially purchased antibodies and other reagents used need to be included in the methods

Figures:
The graphs should be displayed in dot plots and not bar plots
Data availability:
Accession number should be given. Raw RNA-seq (FASTAQ) read files should also be submitted to a database like ENA or NCBI

Reviewer 2

Advance summary and potential significance to field

The manuscript from Cardenas-Rodriguez, et al, “Genetic compensation for cilia defects in cep290/NPHP6 mutants by upregulation of cilia associated small GTPases” describes their use of zebrafish as a model system to study the effects of acute vs prolonged knockdown/deletion of Cep290 and effects on multiple tissues. They find differences in the severity of phenotypes affecting the spine and kidneys depending on whether acute deletions are introduced via morpholinos or permanent changes in the genome resulting from CRISPR/Cas9 mutations resulting in maternal zygotic (MZ) changes. These differences were studied morphologically but also used to generate RNA-seq datasets to look for potential compensation via changes in expression of other genes. Among the genes identified in this screen were those directing expression of three functionally linked proteins involved in ciliary traffic; Unc119b, ARL3, and ARL13B. They went on to show that increasing expression of these genes can reduce and in some cases eliminate phenotypes resulting from the loss of Cep290 and that differences were observed in the tissue specificity and extent of these effects.

This is a very clear and well written manuscript that will be of interest to a broad readership, particularly those interested in ciliary biology and the causes of a range of ciliopathies. It highlights with interesting new data (1) the tissue specificity of genetic changes, (2) important phenotypic differences in acute vs prolonged gene loss, and (3) the potential for compensatory changes in other genes, perhaps importantly those acting in a shared pathway, to alleviate disease causing phenotypes and the potential for designing such changes in a therapeutic setting. Thus, the

© 2021. Published by The Company of Biologists under the terms of the Creative Commons Attribution License (https://creativecommons.org/licenses/by/4.0/).

data provide new potential mechanisms that others will need to consider when interpreting their own studies from KO/KD of cilia associated genes.

Comments for the author

I have several fairly minor suggestions for ways to further improve this manuscript and one rather strong request.

(1) Please explain the large increases in mRNA shown in Fig. 4D in adult eye and kidney for Ex16-17 -/-. Nothing is said in the text and these changes are huge and appear to warrant some description or details.

(2) Confirming the increased abundance of Unc119b, ARL13B, ARL3 via immunoblot would further strengthen the data coming from qPCR and it would be interesting to know if protein levels are increased comparable to their messages. On the plus side, these changes in mRNA (2.3-4.3) are larger than what I am used to seeing (<2.0 fold) so believable but it is the protein that is doing the work here and I am sure that antibodies are available for these targets.

(3) Please elaborate/explain the sentence on p. 16, “Also, upregulation of arl3, arl13b and unc119b is specific to cep290 mutants since we did not observe their upregulation in other zebrafish cilia mutant lines.”

(4) Typo on p. 18, “Pde6e” should be Pde6d (or delta), unless it has a different name in zebrafish.

(5) The data and short discussion in Results and Discussion of Sstr3-GFP doesn’t seem to add much to the rest of the story and is not completely clear why it is included. It could be removed without much loss, though also makes a minor point so could be retained without concerns.

(6) Now to my strong request. That is to please include the full datasets of your RNA-seq screens. You indicate close to 10% of the proteome (~2,000 genes) showed >2-fold increases yet you followed up on only 3. And we don’t know where any of these 3 fit amongst all the hits as far as magnitude of their changes. I realize that some reviewers/editors/readers may decrease enthusiasm for the significance of the three genes you chose to pursue when faced with such a large collection but those data are likely of value to others, for any of a number of possible reasons and I believe all such data should be shared upon publication of their first use. Clearly, it is up to the handling editor to decide the importance of this request, but I believe it should be mandatory in all such cases; though clearly is not today.

First revision

<u>Author response to reviewers' comments</u>

Reviewer 1 Comments for the Author:

Expand the on the human syndromes and associated phenotypes CEP290 LOF mutations causes. A few lines are needed to explain the difference between primary, multi and motile cilia and whether cep290 is specifically localizing to that.

> We thank the reviewer for this suggestion. The introduction has been edited to distinguish for the reader the differences between motile and sensory cilia and the impact of CEP290 mutations in human ciliopathy syndromes.

In the results, please elaborate a bit more why exon 25 and exon 42 were selected for morpholino knockdown. Why was exon 16 selected for CRISPR/Cas9 mutagenesis?

> The results have been edited to better detail the rationale for how the *cep290* gene was

© 2021. Published by The Company of Biologists under the terms of the Creative Commons Attribution License (https://creativecommons.org/licenses/by/4.0/).

targeted:

"We used three different antisense morpholinos targeting the ATG translation initiation codon, and previously published morpholinos targeting the exon 25 splice donor, (Fig 1) and exon 42 splice donor (Fig S1) to acutely disrupt *cep290* expression. These exons were targeted since misplicing generated out of frame exon skipping, predicted to introduce stop codons and premature translation termination.....Also we generated a stable *cep290* mutant using CRISPR/Cas9 where a 10bp deletion in exon 16 causes a frameshift and premature termination codon (PTC, Fig 1B). Exon 16 was targeted based on high efficiency Cas9 cutting and introduction of N-terminal out of frame stop codons."

In results, discussing figure 5B highlight ('revealed a set 1,953 genes upregulated > 2-fold in MZcep290-/- mutants') where the 1953 genes were 2 fold change was derived from. There are no supplemental data files (Excel file) with the baseline and differential expression data, so I am not in the position to check this claim. This will need to be supplied in the revisions.

We apologize for the oversight of not submitting the gene list tables with the manuscript. These are now included as Supplemental data files Tables 1-3 with differentially expressed genes in mutants vs. wildtype and mutants vs. morphants. Raw and processed data files have also been submitted to NCBI GEO under accession GSE175491.

I encourage the authors to find a way to display the gene ontology analysis (i.e. GOrilla, STRING Network analysis, KEGG etc.) and publish it as a supplemental figure and make the data files associated with these analyses available.

Genes upregulated in MZcep290 mutants were analyzed by DAVID functional annotation. The gene enrichment sets are noted in the revised results section and now detailed in Supplemental Table 4.

It is strongly encouraged to perform a morpholino (cep290ex25 and control) titration experiment where e.g. half and a quarter of the original dosages are injected to determine ciliopathy frequency and subsequent protein expression analysis (i.e. 50% ex25 + 50%ctrlMO; 25%ex25 +75%ctrlMO mixtures). This to determine when morpholino and mutant would display a similar phenotype and it would provide information on when the compensatory mechanism kicks in.

We thank the reviewer for this interesting suggestion. However, since heterozygote *cep290* mutants show no overt phenotype it is not clear to us what inducing a partial loss of function or gene dosage using morpholinos would yield in terms of mechanistic information. Since both MZcep290 and cep290 MO knockdowns appear to cause a full loss of full length Cep290 protein, we feel the compensatory induction of GTPases may have more to do with the timing and duration of cep290 deficiency than the dose. We do note the data of Lessieur et al in the discussion on the impact of cep290 heterozygosity on arl13b mutants showing that reduction in cep290 gene dosage can impact other loss of function phenotypes.

This could be further bolstered with co-injecting RNAs the arl and unc mRNAs and/or qPCR expression analysis. A morpholino knockdown of unc119b and arl13b in the mutants should induce a worse phenotype as it disrupts the compensatory mechanism (if there is a genetic interaction). This is a recommended experiment and if it is not feasible it should be discussed in the discussion as a future directions statement.

This is an interesting idea that we did not pursue. In support of the reviewers idea, we discuss the work of Lessieur et al. who have shown that *cep290* heterozygosity worsens an *arl13b* mutant phenotype in zebrafish. However since *arl13b* mutation has a similar and stronger phenotype than *cep290* loss of function, it may be difficult to distinguish the contribution of *cep290* deficiency to ciliopathy in a full *arl13b* knockdown background. Hence we chose not to pursue this line of experimentation. We added a sentence in the discussion to acknowledge that full double loss of function could possibly show stronger ciliopathy phenotypes.

© 2021. Published by The Company of Biologists under the terms of the Creative Commons Attribution License (https://creativecommons.org/licenses/by/4.0/).

Discussion:

Add a couple of sentences to discuss / summarize the correlation and discrepancies of the zebrafish mutant phenotype and human/mouse phenotypes.

We have added two sentences at the start of the discussion to emphasize the variable expressivity of CEP290 mutations.

The authors discuss sclc25a46 and CBPA, there are also studies of golgb1 knockdown/mutant compensation via glycosyltransferases and RCAN2 (PMID: 29643119; PMID: 29093022; PMID: 28546340). These should be added.

We thank the reviewer for pointing out these interesting and highly relevant papers. We have highlighted this work in the discussion. Compensation of giantin loss of function really mirrors what we see with cep290 and it may be relevant that both proteins are involved in facilitating membrane protein transport.

Arl13b is known to regulate vangl2 and wnt signaling planar cell polarity pathway, could a disruption of this be repaired due to overexpression and hence the reduction of the ciliopathy phenotype in rescue experiments? This needs to be discussed (PMID: 27571019 already cited; PMID: 20305649)

While this is an interesting idea, we don't have evidence for planar polarity phenotypes in *cep290* mutants or morphants. Given this, it's unlikely that planar polarity per se underlies the photoreceptor or KV phenotypes we do observe in *cep290* mutants or morphants. There is currently no data linking *cep290* and *vangl2* although it may be worth a look. So while it may be an interesting avenue for future investigation, we feel it would be rather speculative to add this idea to the discussion and we choose not to raise this concept.

Material methods:

Statistical methods should be provided in a separate heading including software used to calculate p-values RNA- seq analysis needs to be elaborated on which illumina platform and what bioinformatics pipeline was used to map the reads onto the zebrafish genome and how differential expression was calculated Catalogue numbers of commercially purchased antibodies and other reagents used need to be included in the methods

We have revised the materials and methods to include the requested details and statistical analyses used.

Figures:
The graphs should be displayed in dot plots and not bar plots.

We remade all bar graphs as dot plots.

Data availability:

Accession number should be given. Raw RNA-seq (FASTAQ) read files should also be submitted to a database like ENA or NCBI

All RNAseq raw and processed data has been deposited in NCBI GEO under accession GSE175491.

Reviewer 2 Comments for the Author:

(1) Please explain the large increases in mRNA shown in Fig. 4D in adult eye and kidney for Ex16-17 -/-. Nothing is said in the text and these changes are huge and appear to warrant some description or details.

© 2021. Published by The Company of Biologists under the terms of the Creative Commons Attribution License (https://creativecommons.org/licenses/by/4.0/).

Thanks for pointing this out. The RTPCR results are presented as fold change relative to control wild type mRNA samples. In brief, wildtype primers for the targeted Crispr mutation site don't amplify mutant mRNA and mutant primers effectively amplify mutant mRNA. This explanation is added to the figure legend:

"(D) RT-qPCR from adult eye and kidney samples. Specific wild type and mutant primers for the targeted exon16 were used to quantify wild type and mutated cep290 mRNA. As expected, wild type primers did not generate a RT-PCR product on mutant mRNA samples relative to wild type mRNA while mutant primers generated significant product on mutant mRNA samples relative to wild type mRNA samples. Primers for ex3-ex5, ex19-ex20 and ex50-52 were used to quantify non-sense mediated decay (NMD)."

(2) Confirming the increased abundance of Unc119b, ARL13B, ARL3 via immunoblot would further strengthen the data coming from qPCR and it would be interesting to know if protein levels are increased comparable to their messages. On the plus side, these changes in mRNA (2.3-4.3) are larger than what I am used to seeing (<2.0 fold) so believable but it is the protein that is doing the work here and I am sure that antibodies are available for these targets.

We agree it would be interesting to know final protein levels for Unc119b, Arl3, and Arl13b in *cep290* mutants. This will become important as we pursue future studies of this phenomenon and establish mechanism of mRNA upregulation. For this manuscript we focused on increased mRNA expression and mimicking that with mRNA injections. Due in part to personnel departure and pandemic restrictions we are limited in resources to take on new experiments right now. Nonetheless we acknowledge the importance of the reviewers comment and will take on studies of protein levels in future work.

(3) Please elaborate/explain the sentence on p. 16, "Also, upregulation of arl3, arl13b and unc119b is specific to cep290 mutants since we did not observe their upregulation in other zebrafish cilia mutant lines."

To clarify this sentence we reworded it: " Compensation of ciliopathy by upregulation of *arl3, arl13b and unc119b* appears to be specifically induced by mutation in the *cep290* gene since we did not observe upregulation of these genes in the cilia motility mutant *smh* (*ccdc103*) or the IFT mutant *oval* (*ift88*)."

(4) Typo on p. 18, "Pde6e" should be Pde6d (or delta), unless it has a different name in zebrafish.

Thanks, fixed.

(5) The data and short discussion in Results and Discussion of Sstr3-GFP doesn't seem to add much to the rest of the story and is not completely clear why it is included. It could be removed without much loss, though also makes a minor point so could be retained without concerns.

The idea of that experiment was to test the relationship between cilia axoneme length and cilia membrane delivery. One of the points in our paper is that Cep290 deficiency in zebrafish specifically affects cilia where Cep290 is localized at pericentriolar satellites (a site of membrane cargo delivery) and mutation results in the accumulation of membrane vesicles there. The rescue of KV cilia length by arl3/arl13b/unc119b implies to us that bypassing an inefficiency in membrane delivery by catalyzing release and delivery of cargo affects axoneme length. It is still somewhat mysterious how this works. How is the amount of cilia membrane sensed by the IFT machinery to add more tubulin to the tips of axonemes? The Sstr3-GFP experiment is included to show that multiple ways of driving membrane into cilia can restore length by a kind of mass action of ectopic cilia-targeted membrane production. As long as the reviewer can see this point I prefer to report this result.

We modified the statement in the discussion to try to clarify this rationale for the experiment.

© 2021. Published by The Company of Biologists under the terms of the Creative Commons Attribution License (https://creativecommons.org/licenses/by/4.0/).

(6) Now to my strong request. That is to please include the full datasets of your RNA-seq screens. You indicate close to 10% of the proteome (~2,000 genes) showed >2-fold increases yet you followed up on only 3. And we don't know where any of these 3 fit amongst all the hits as far as magnitude of their changes. I realize that some reviewers/editors/readers may decrease enthusiasm for the significance of the three genes you chose to pursue when faced with such a large collection but those data are likely of value to others, for any of a number of possible reasons and I believe all such data should be shared upon publication of their first use. Clearly, it is up to the handling editor to decide the importance of this request, but I believe it should be mandatory in all such cases; though clearly is not today.

> We regret the oversight of not including this data in our first submission; primary RNAseq data has been submitted to NCBI GEO under accession GSE175491. We also include in this revision new supplemental tables on the differential gene expression analysis from RNAseq and the Functional annotation analysis that highlighted small GTPases as an enriched functional cluster in the *cep290* mutant RNAseq.

We thank the reviewers for the time and effort they put into making this a stronger manuscript. We hope the changes made will clarify our work and make it acceptable to the Journal of Cell Science.

Second decision letter

MS ID#: JOCES/2021/258568

MS TITLE: Genetic compensation for cilia defects in cep290/NPHP6 mutants by upregulation of cilia-associated small GTPases

AUTHORS: Magdalena Cardenas-Rodriguez, Christina Austin-Tse, Judith G.M Bergboer, Elisa Molinari, Yuya Sugano, Ruxandra Bachmann-Gagescu, John A Sayer, and Iain A Drummond
ARTICLE TYPE: Research Article

Thank you for the careful revisions to your manuscript. I am happy to tell you that your manuscript has been accepted for publication in Journal of Cell Science, pending standard ethics checks. I did not find it necessary to return this to the reviewers so no reports are available on this version. I found that your revisions suitably addressed their substantive comments and therefore am happy to accept your paper for publication.

© 2021. Published by The Company of Biologists under the terms of the Creative Commons Attribution License (https://creativecommons.org/licenses/by/4.0/).
